# The FAK inhibitor BI 853520 exerts anti-tumor effects in breast cancer

**DOI:** 10.1038/s41389-018-0083-1

**Published:** 2018-09-20

**Authors:** Stefanie Tiede, Nathalie Meyer-Schaller, Ravi Kiran Reddy Kalathur, Robert Ivanek, Ernesta Fagiani, Philip Schmassmann, Patrick Stillhard, Simon Häfliger, Norbert Kraut, Norbert Schweifer, Irene C. Waizenegger, Ruben Bill, Gerhard Christofori

**Affiliations:** 1 Department of Biomedicine, University of Basel, 4058 Basel, Switzerland; 2 Swiss Institute of Bioinformatics, 4053 Basel, Switzerland; 3 Boehringer Ingelheim RCV GmbH & Co KG, 1121 Vienna, Austria

## Abstract

Focal adhesion kinase (FAK) is a cytoplasmic tyrosine kinase that regulates a plethora of downstream signaling pathways essential for cell migration, proliferation and death, processes that are exploited by cancer cells during malignant progression. These well-established tumorigenic activities, together with its high expression and activity in different cancer types, highlight FAK as an attractive target for cancer therapy. We have assessed and characterized the therapeutic potential and the biological effects of BI 853520, a novel small chemical inhibitor of FAK, in several preclinical mouse models of breast cancer. Treatment with BI 853520 elicits a significant reduction in primary tumor growth caused by an anti-proliferative activity by BI 853520. In contrast, BI 853520 exerts effects with varying degrees of robustness on the different stages of the metastatic cascade. Together, the data demonstrate that the repression of FAK activity by the specific FAK inhibitor BI 853520 offers a promising anti-proliferative approach for cancer therapy.

## Introduction

Every year, more than 1.4 million women worldwide are diagnosed with breast cancer, and over 450,000 women will lose their lives to this disease, mostly due to metastasis^1^. Over the past decades, we have gained many important insights into breast cancer biology, which in turn have allowed the development of therapeutic approaches targeting molecules and signaling pathways specifically present in breast cancer cells^2,3^. Previous studies have linked the overexpression and activation of focal adhesion kinase (FAK) with the initiation and progression of a wide variety of malignancies, such as ovarian, head and neck, and breast carcinoma^2–6^. FAK is a multifunctional cytoplasmic tyrosine kinase that forms an important component of focal adhesion sites^7–11^. Once recruited by signals initiated at integrin-mediated extracellular matrix attachment sites and by multiple growth factor receptors, such epithelial growth factor receptor, vascular endothelial growth factor receptor, and platelet-derived growth factor receptor, FAK undergoes a conformational change, enabling autophosphorylation of the tyrosine residue (Y) 397 at its N-terminal domain^3,12,13^. Subsequently, phosphorylated Y397 serves as a docking site for SRC homology 2 containing SRC family kinases, which results in a fully active FAK-SRC signaling complex that can trigger various downstream signaling pathways known to control cell migration, invasion, proliferation, and death—all activities pivotal for malignant tumor progression^3,7,10,11,14–18^.

Previous studies have indicated that the forced expression of FAK in endothelial cells enhances angiogenesis and that the ectopic expression of a constitutive-active form of FAK in murine mammary cancer cells promotes their proliferation. Conversely, decreasing FAK expression impairs cancer cell proliferation in vitro and in vivo^6,10,19–21^ and inhibits endothelial cell proliferation in vitro and in vivo. These data together suggest a linear relationship between FAK activity and tumorigenesis^8,19,20,22,23^. However, a recent study has reported that the heterozygous depletion of FAK in endothelial cells increases endothelial cell proliferation and tumor angiogenesis, indicating a non-linear effect of FAK activity in carcinogenesis^3,19,20^. Supporting this notion, low-dose treatment with the FAK inhibitor (FAK-I) PF-573228 increases microvessel sprouting ex vivo and tumor growth in vivo^19^. These results indicate that the causal link between FAK activity and tumor progression still escapes a final conclusion, and further investigations are warranted to delineate the functional contribution of FAK to carcinogenesis.

We have evaluated the therapeutic and biological effects of BI 853520, a novel, potent, and selective small chemical entity kinase FAK-I^24^, in cultured murine breast cancer cells in vitro and in various transplantation and transgenic mouse models of breast cancer in vivo. Gene expression profiling of primary tumors of mice treated with BI 853520 reveals a decrease in the expression of genes regulating cell proliferation. Indeed, treatment with BI 853520 provokes a significant reduction in cell proliferation in vitro and in vivo. In contrast, BI 853520 exerts heterogeneous effects on pulmonary metastasis at different levels of the metastatic cascade depending whether it is used in a neoadjuvant or adjuvant therapeutic setting. Thereby, the epithelial cell adhesion molecule E-cadherin may serve as a potential predictive marker for increased sensitivity of cancer cells to treatment with BI 853520.

## Results

### The FAK-I BI 853520 represses Y397-FAK autophosphorylation

**Fig. 1:**
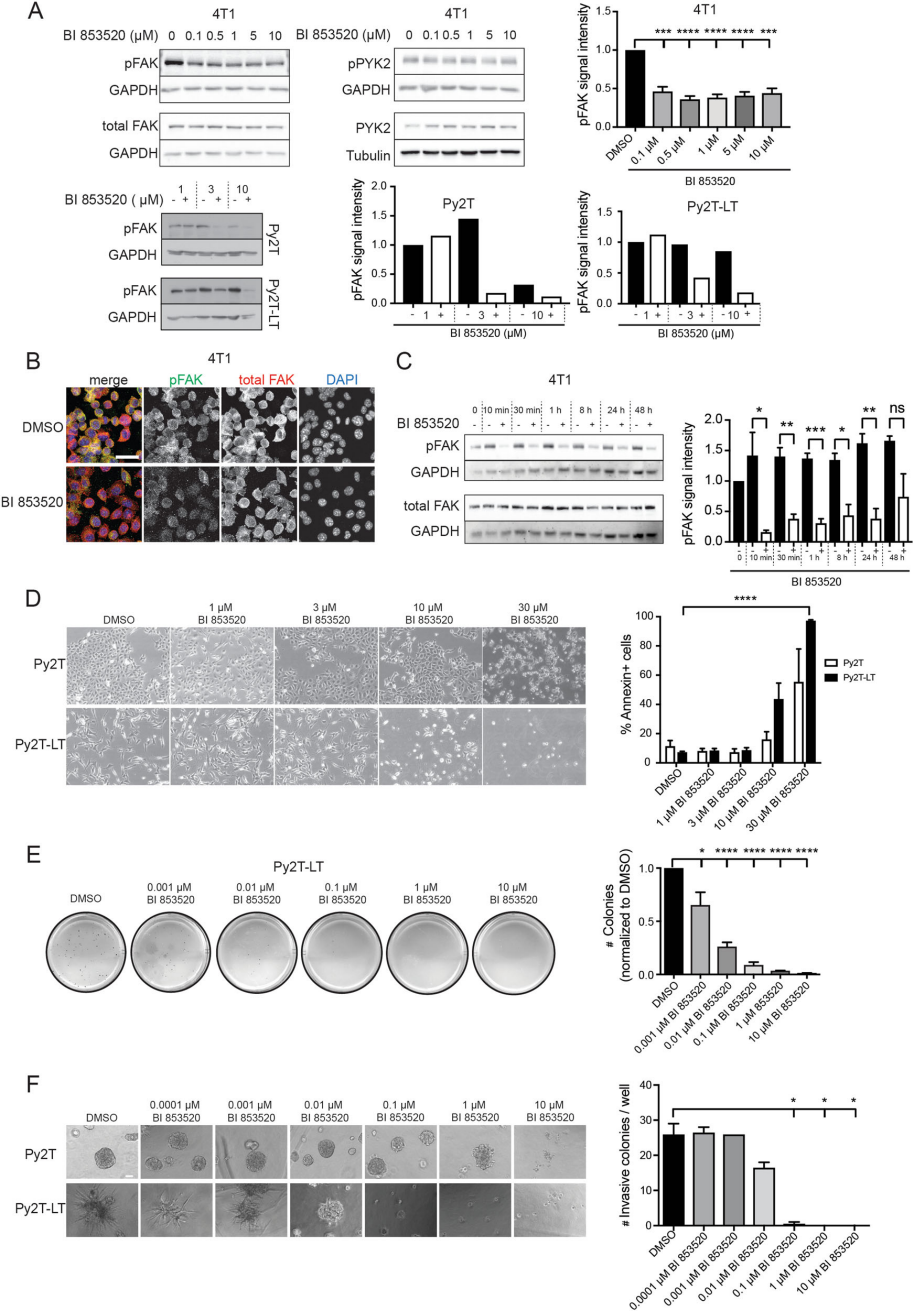
BI 853520 decreases Y397-FAK phosphorylation in a dose-dependent and time-dependent manner. **a** To determine the effective dose of BI 853520, 4T1, Py2T, and Py2T-LT cells were exposed to increasing concentrations of BI 853520 (0, 0.1, 0.5, 1, 5, and 10 µM for 4T1 cells; 1, 3, and 10 µM for Py2T and Py2T-LT cells) for 24 h. Top left panel: 4T1 cell lysates were examined by immunoblotting analysis for phospho-FAK-Y397 (pFAK) and total FAK (FAK). GAPDH served as a loading control (*n* = 4). Immunoblotting for phospho-PYK2-Y402 (pPYK2), total PYK2 (PYK2), and GAPDH and tubulin as loading control is shown in the top middle panel (*n* = 2). Top right panel: Quantification of pFAK signal intensity normalized to GAPDH and total FAK/GAPDH ratios from the immunoblotting analysis shown on the left. Statistical analysis was performed using an unpaired, two-tailed Student’s *t* test. ****p < 0.0001, ***p < 0.001. Bottom left panel: Py2T and Py2T-LT cell lysates were examined by immunoblotting analysis for phospho-FAK-Y397 (pFAK). GAPDH served as a loading control (*n* = 1). Right panel: Quantification of pFAK signal intensity normalized to GAPDH from the immunoblotting analysis shown on the left. **b** 4T1 cells grown on coverslips were treated with the effective dose of BI 853520 (0.1 µM) for 96 h, followed by immunofluorescence staining for pFAK (Y397, green), total FAK (red), and DNA (blue). Images were obtained using a laser-scanning confocal microscope Leica SP5 (*n* = 2). Scale bar, 30 µm. **c** To determine the onset and duration of BI 853520-mediated pFAK downregulation, 4T1 cells were treated for the times indicated with 0.1 µM BI 853520. Cell lysates were analyzed by immunoblotting for pFAK (Y397), total FAK, and GAPDH as a loading control (*n* = 3). Right panel: Quantification of pFAK signal intensity normalized to GAPDH and total FAK/GAPDH ratios from the immunoblotting analysis shown on the left. Statistical analysis was performed using an unpaired, two-tailed Student’s *t* test. ***p < 0.001, **p < 0.01, and *p < 0.05. **d** Cytotoxic effects of high doses of BI 853520 on murine breast cancer cells cultured on plastic surface. Epithelial Py2T and mesenchymal Py2T-LT murine breast cancer cells were treated with BI 853520 at the concentrations indicated and phase contrast micrographs were taken. Cells were then suspended, and staining for Annexin-V as marker of apoptosis was quantified by flow cytometry (*n* = 2). Statistical analysis was performed using an unpaired, two-tailed Student’s *t* test. ****p < 0.0001. Scale bar: 120 μM. **e** BI 853520 represses colony formation in three-dimensional growth conditions. Py2T-LT cells were seeded in soft agar gels and treated with increasing concentrations of BI 853520 as indicated. The numbers of colonies were quantified. (*n* = 3). Unpaired, two-tailed Student’s *t* test. ****p < 0.0001, *p < 0.05. **f** BI 853520 represses mesenchymal murine breast cancer cell invasion cultured in Matrigel. Epithelial Py2T and mesenchymal Py2T-LT murine breast cancer cells were seeded in Matrigel and treated with increasing concentrations of BI 853520 as indicated. Phase contrast microscopy pictures were taken and the numbers of invasive colonies were quantified. Epithelial Py2T cells formed differentiated spheres which only responded to BI 853520 at high, toxic concentrations (*n* = 1). Statistical analysis was performed using an unpaired, two-tailed Student’s *t* test. *p < 0.05. Scale bar: 60 μM. ns not significant

To determine the in vitro efficacy of the FAK-I BI 853520 in repressing Y397-FAK phosphorylation in differentiated breast cancer cells and in breast cancer cells that have undergone an epithelial–mesenchymal transition (EMT) and also to test the generality of the findings, we employed a combination of various murine mammary cancer cell lines. 4T1 cells, which have been derived from a spontaneous tumor in a mammary gland of a Balb/c mouse, show hallmarks of a partial EMT and are highly metastatic upon transplantation into mice. The Py2T cell line has been established from a tumor of a MMTV-PyMT transgenic mouse. Py2T cells exhibit an epithelial cell morphology and undergo a reversible EMT upon long-term treatment with transforming growth factor-β (TGFβ) in vitro (Py2T-LT cells)^25^. 4T1, Py2T, and Py2T-LT cells were treated with different concentrations of BI 853520, and Y397-FAK phosphorylation was assessed by immunoblotting analysis and immunofluorescence staining using a phospho-Y397-FAK-specific antibody. BI 853520 significantly reduces Y397-FAK autophosphorylation in all cell types (Fig. 1a, b), while the phosphorylation levels of FAK’s homolog PYK2 remained unaffected up to micromolar concentrations (Fig. 1a). Next, a time-course experiment was performed on 4T1 cells treated for various time points with 0.1 µM BI 853520 to analyze FAK-I efficacy in a time-dependent manner. Decreased Y397-FAK autophosphorylation following 0.1 µM BI 853520 treatment occurred within 10 min and was substantially reduced at least for the following 48 h (Fig. 1c), demonstrating a fast and potent inhibition of FAK by BI 853520 in this highly metastatic murine breast cancer cell line.

### BI 853520 represses tumor cell proliferation and invasion only in 3D culture

We next assessed the repressive effect of the FAK-I BI 853520 on proliferation and invasion of 4T1 cells, Py2T cells and Py2T cells that have undergone an EMT (Py2T-LT). When cultured in two-dimensional (2D) plastic dishes, epithelial Py2T cells and mesenchymal Py2T-LT cells did not show any change in cell morphology or proliferation upon treatment with up to 3 µM BI 853520. However, at a concentration of 10 µM and above a cytotoxic effect, a massive apoptosis could be observed with both cell types (Fig. 1d).

To further investigate the repressive effect of BI 853520 on breast cancer cell proliferation in vitro, we conducted an MTS assay on 4T1, Py2T, and Py2T-LT cells as previously reported (Suppl. Figure 1A)^26^. Increasing concentrations of BI 853520 led to a modest yet significant reduction in cell viability of all three breast cancer cell types grown in 2D already at 10–100 nM, with a massive reduction at the toxic concentrations above 3 μM. Flow cytometry-based 5-ethynyl-2′-deoxyuridine/propidium iodide (EdU/PI) cell cycle analysis of 4T1 cells revealed a shift in cells leaving the S phase and entering the G1 phase (Suppl. Figure 1B). Consistent with these results, the percentage of phospho-histone 3 (pH3)-positive nuclei was decreased in 4T1 cells with increasing concentrations of BI 853520, while increased rates of apoptosis were only observed at the toxic concentration of 10 μM BI 853520 (Suppl. Figure 1C, D).

In contrast, when cultured in a 3D environment, such as in soft agar, colony formation of mesenchymal Py2T-LT started to be compromised already at a concentration of 1 nM BI 853520 (Fig. 1e). Epithelial Py2T cells did not form colonies in this assay even in the absence of BI 853520 (data not shown).

We have previously reported that mesenchymal Py2T-LT cells show a massive invasion into the surrounding matrix when cultured in 3D in Matrigel, while epithelial Py2T cells form highly differentiated spheres^25^ (Fig. 1f and Suppl. Figure 1E). Comparable invasive growth was also observed with 4T1 cells when cultured in Matrigel (Suppl. Figures 1E, 2A). The invasion of mesenchymal Py2T-LT cells was already inhibited at 10 nM BI 853520, while sphere formation of epithelial Py2T cells was only affected by increased cell death at the toxic concentrations of BI 853520 higher than 1 μM (Fig. 1f). This effect was not as pronounced with 4T1 cells cultured in Matrigel, where BI 853520 at doses above 0.1 μM repressed cell invasion, although without statistical significance, while high doses exerted a toxic effect (Suppl. Figure 1E). The efficient repression of FAK autophosphorylation in the 3D Matrigel cultures was determined by immunofluorescence staining of the spheroids and by immunoblotting of spheroid lysates (Suppl. Figure 2A, B). Despite a decrease in cell invasion, the immunofluorescence and immunoblotting analysis revealed that FAK inhibition did not substantially affect the expression of the mesenchymal markers fibronectin, vimentin, or Zeb1 and the epithelial markers E-cadherin or ZO1 in the spheroid cultures.

These results indicate that the specific inhibition of cell proliferation and invasion at low doses of BI 853520 is functional only in three-dimensional cell culture conditions, whereas cells cultured on plastic only respond to BI 853520 at very high, toxic doses of BI 853520.

### BI 853520 attenuates primary tumor growth

To examine the repressive effect of the FAK-I BI 853520 on primary tumor growth in vivo, we have employed a variety of cellular and transgenic and transplantation mouse models of breast cancer. MMTV-PyMT transgenic mice develop mammary gland tumors and recapitulate the stepwise progression from differentiated mammary gland epithelia to hyperplasia, adenoma, carcinoma, and lung metastasis^27^. Py2T murine epithelial breast cancer cells are highly tumorigenic and gain mesenchymal characteristics when implanted into mice in vivo, yet they are rarely metastatic^25^. 4T1 cells are highly metastatic breast cancer cells derived from a spontaneous mammary gland tumor of a Balb/c mouse^28^. Finally, E-cadherin-proficient MTflECad cells have been isolated from a lymph node metastasis of a MMTV-Neu;*Cdh1*fl/fl tumor-bearing mouse and have been infected with an adenovirus expressing Cre recombinase to obtain the E-cadherin-deficient cell line MTΔECad^29^.

**Fig. 2:**
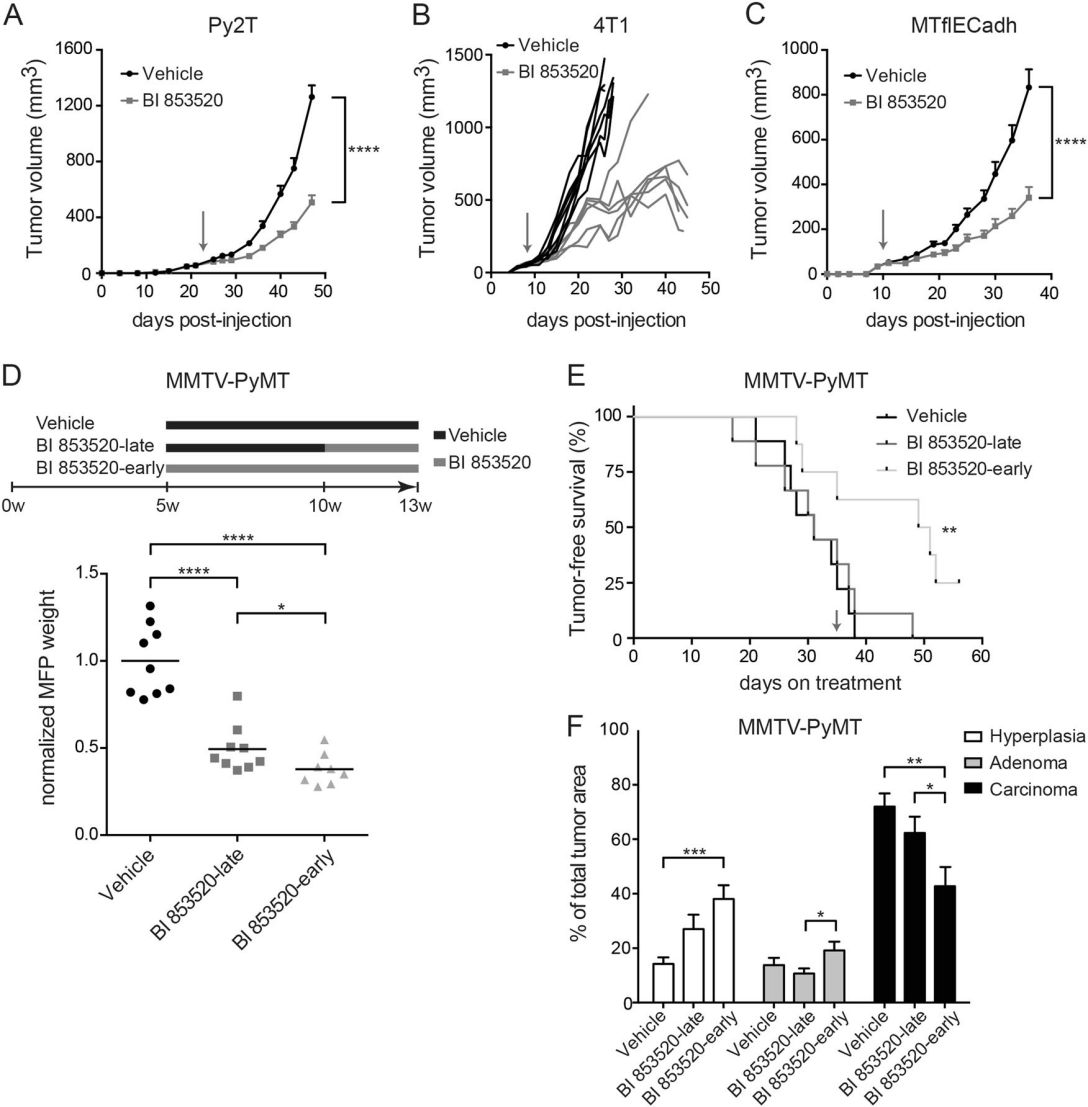
BI 853520 decreases primary tumor growth in various orthotopic pre-clinical mouse models of breast cancer. **a** Oral treatment of mice bearing Py2T breast tumors with daily 50 mg/kg BI 853520 dissolved in Natrosol or with Natrosol-vehicle alone from day 22 post injection (indicated by an arrow) for 25 consecutive days significantly decreased tumor volume over time. *n* = 10 mice per treatment cohort. **b** Oral treatment of mice bearing 4T1 breast tumors with daily 50 mg/kg BI 853520 dissolved in Natrosol or with Natrosol-vehicle alone from day eight post injection (indicated by an arrow) significantly decreased tumor volume over time. Tumor growth curves of individual mice are shown. Vehicle cohort, *n* = 7 mice; BI 853520 cohort, *n* = 6 mice. **c** Oral treatment of mice bearing MTflECad breast tumors with daily 50 mg/kg BI 853520 dissolved in Natrosol or with Natrosol-vehicle alone from day 10 post injection significantly decreased tumor volume over time (indicated by an arrow). *n* = 9 mice per treatment cohort. **d** Top: Oral treatment schedule for the treatment of MMTV-PyMT transgenic mice with vehicle, BI 853520-late (50 mg/kg daily, from 10 to 13 weeks of age) or BI 853520-early (50 mg/kg daily, from 5 to 13 weeks of age). Bottom: Normalized mammary fat pad (MFP) weights for each treatment cohort. All mice were sacrificed at 13 weeks of age, and primary tumors and lungs were used for further analysis. Vehicle cohort, *n* = 9; BI 853520-late, *n* = 9; BI 853520-early, *n* = 8. **e** Early BI 853520 treatment significantly improves tumor-free survival of MMTV-PyMT mice. Days on treatment starting from week 5 of age are shown. The time reaching termination criteria was determined for the mice treated as described in **d**. As shown here, at the time point of treatment initiation of the BI 853520-late group (arrow; day 35 on treatment), most mice in this group displayed palpable tumors. **f** Grading of primary tumors of MMTV-PyMT transgenic mice treated as described in **d**. Data are shown as the percentage of the average area covered by each grade of two histological sections per mouse. Early BI 853520 treatment delays malignant tumor progression (increase in hyperplasia and adenoma stage, decrease in carcinoma stage). Statistical analysis was performed by unpaired, two-tailed Mann–Whitney *U* test (**a**, **c**, **d**), by log-rank (Mantel–Cox) test (**e**), and unpaired, two-tailed Student’s *t* test (**f**). All data are depicted as means ± SEM. ****p < 0.0001, ***p < 0.001, **p < 0.01, *p < 0.05

Py2T, 4T1, or MTflECad breast cancer cell lines were orthotopically transplanted into the mammary fat pad of syngeneic FVB/N, Balb/c, or immunodeficient nude (nu/nu) mice, respectively, and mice were treated with 50 mg/kg BI 853520 by oral gavage. BI 853520 treatment significantly suppressed primary tumor growth of all three cell lines in vivo (Fig. 2a–c and Suppl. Figure 3A, B). Together, the data suggest a model-independent suppression of primary tumor growth by BI 853520 which is consistent with data shown by Hirt et al^30,31^. Consistent with the results from experiments with cultured cell lines in vitro (Fig. 1a, c), immunoblotting analysis of primary Py2T tumors of mice treated with BI 853520 for 3 days displayed a decrease in the levels of Y397-FAK phosphorylation (Suppl. Figure 3C). Similarly, Y397-FAK phosphorylation levels were reduced, although not with statistical significance, when mice bearing 4T1 tumors were treated with a single dose of BI 853520 and sacrificed 1, 4, or 8 h post treatment (Suppl. Figure 3D).

Since transplantation models of cancer cell lines lack the multi-step development of tumors as observed in patients, we next tested the effect of BI 853520 on primary tumor growth and progression in the MMTV-PyMT transgenic mouse model of metastatic breast cancer, a valuable tool to investigate metastatic breast cancer development and progression^27^. Strikingly, both the administration of BI 853520 at an early stage of tumor development (5 weeks of age; “BI 853520 early”) and at 10 weeks of age (“BI 853520 late”), a time point when most MMTV-PyMT mice had palpable tumors, significantly reduced mammary fat pad weight as a surrogate endpoint for total tumor burden per mouse (Fig. 2d). Notably, administration of BI 853520 at 5 weeks of age significantly delayed the development of palpable tumors, that is, tumor-free survival (Fig. 2e). In addition, BI 853520 inhibited tumor progression by reducing the formation of invasive carcinomas within the primary tumors of the transgenic mice (Fig. 2f). In summary, the FAK-I BI 853520 exerts a potent capability to repress primary tumor growth and tumor progression in multiple mouse models of breast cancer.

### BI 853520 restrains tumor cell proliferation

**Fig. 3:**
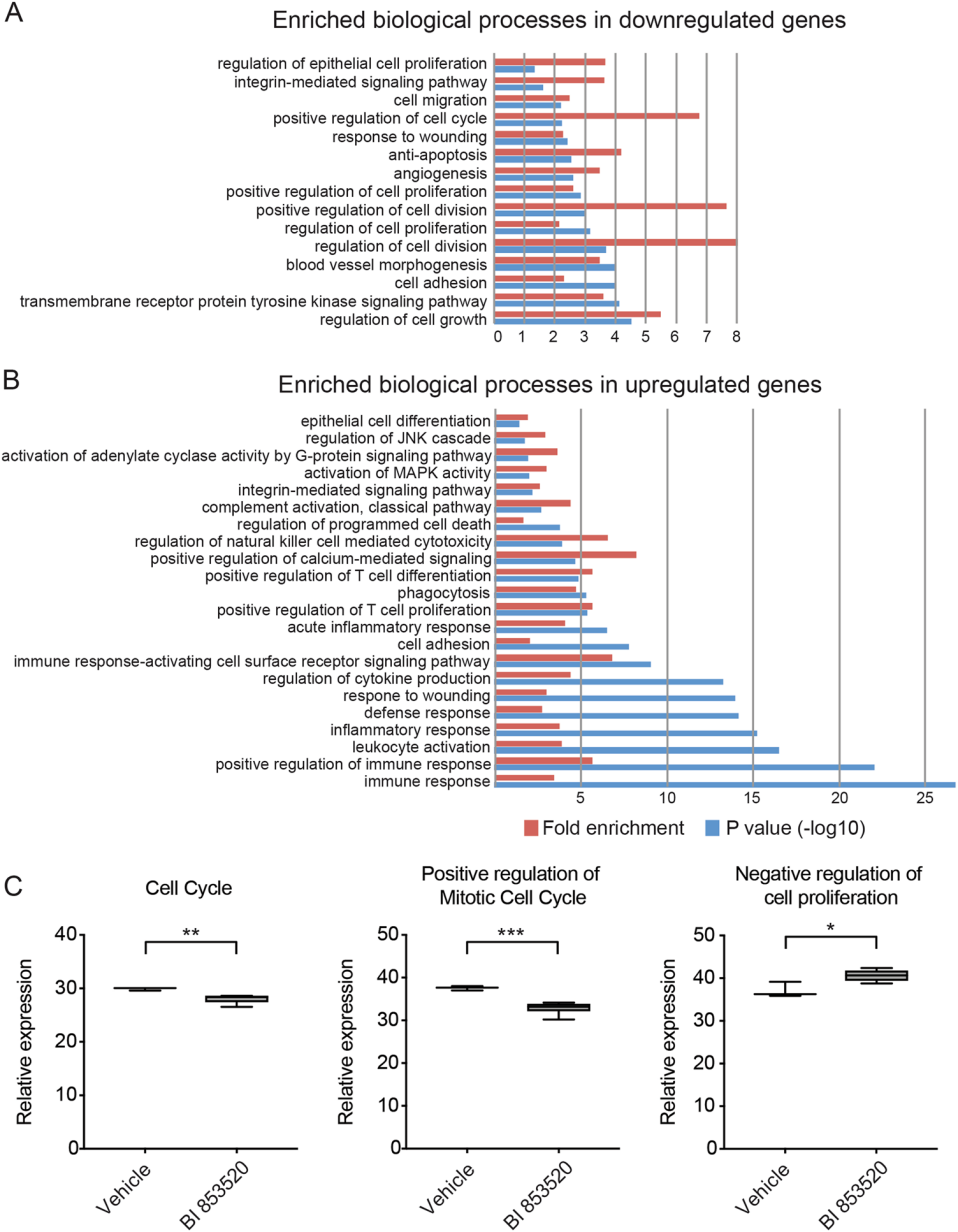
BI 853520 treatment downregulates genes implicated in cell proliferation and upregulates genes implicated in immune response. **a**, **b** List of biological processes enriched in downregulated and upregulated genes, respectively, upon treatment of mice bearing primary 4T1 tumors with 50 mg/kg BI 853520 dissolved in Natrosol-vehicle or vehicle alone for 5 days. All terms shown in the bar graphs are statistically significant (*p* value ≤0.05). The blue and red bars represent *p* values in −log 10 format and the fold enrichment for each term, respectively. The *p* values and enrichment were computed using Fisher’s exact test. **c** Box whisker plots showing the relative expression of genes involved in processes of cell cycle, positive regulation of mitotic cell cycle, and negative regulation of cell proliferation that were identified using gene set enrichment analysis. Relative gene expression was compared between vehicle-treated group (*n* = 3) and BI 853520-treated group (*n* = 6). The significance between the groups is shown as *p* values using an unpaired, two-tailed Student’s *t* test. ***p < 0.001, **p < 0.01, and *p < 0.05

In order to delineate the molecular mechanisms leading to the therapeutic effect of BI 853520 on primary tumor growth, mice harboring 4T1 primary tumors were treated for 5 days with BI 853520, and RNA extracted from total tumors was subjected to next-generation sequencing. Only primary tumors with sufficient RNA quality were included into further analysis (Suppl. Figure 4A). Gene expression correlation analysis displayed a clear separation of transcriptomic profiles derived from primary tumors of mice treated with BI 853520 or vehicle (Suppl. Figure 4B). Comparative gene expression analysis of primary tumors of mice treated with BI 853520 vs. vehicle control revealed 1293 upregulated and 475 downregulated genes (cutoffs: *p* value ≤0.05, fold change ±1.5). Functional enrichment analysis for biological processes and signaling pathways indicated that the regulation of epithelial cell proliferation and positive regulation of cell cycle/cell proliferation/cell division were enriched in genes downregulated by BI 853520 treatment (Fig. 3a). In line with this finding, gene set enrichment analysis confirmed a significant reduction in the relative expression of genes important for cell cycle and positive regulation of mitotic cell cycle (including cyclin-dependent kinase 1 and 4; Cdk1: log 2 fold change = −0.4519, false discovery rate (FDR) = 4.24e−03; Cdk4: log 2 fold change = −0.3072, FDR = 0.003718), while the negative regulation of cell proliferation was increased following BI 853520 treatment (Fig. 3c and Suppl. Figure 4C). Interestingly, biological processes linked to T cell differentiation and cell proliferation, (acute) inflammatory response, cytokine production, and leukocyte activation were enriched in the group of genes upregulated by BI 853520 treatment (Fig. 3b).

**Fig. 4:**
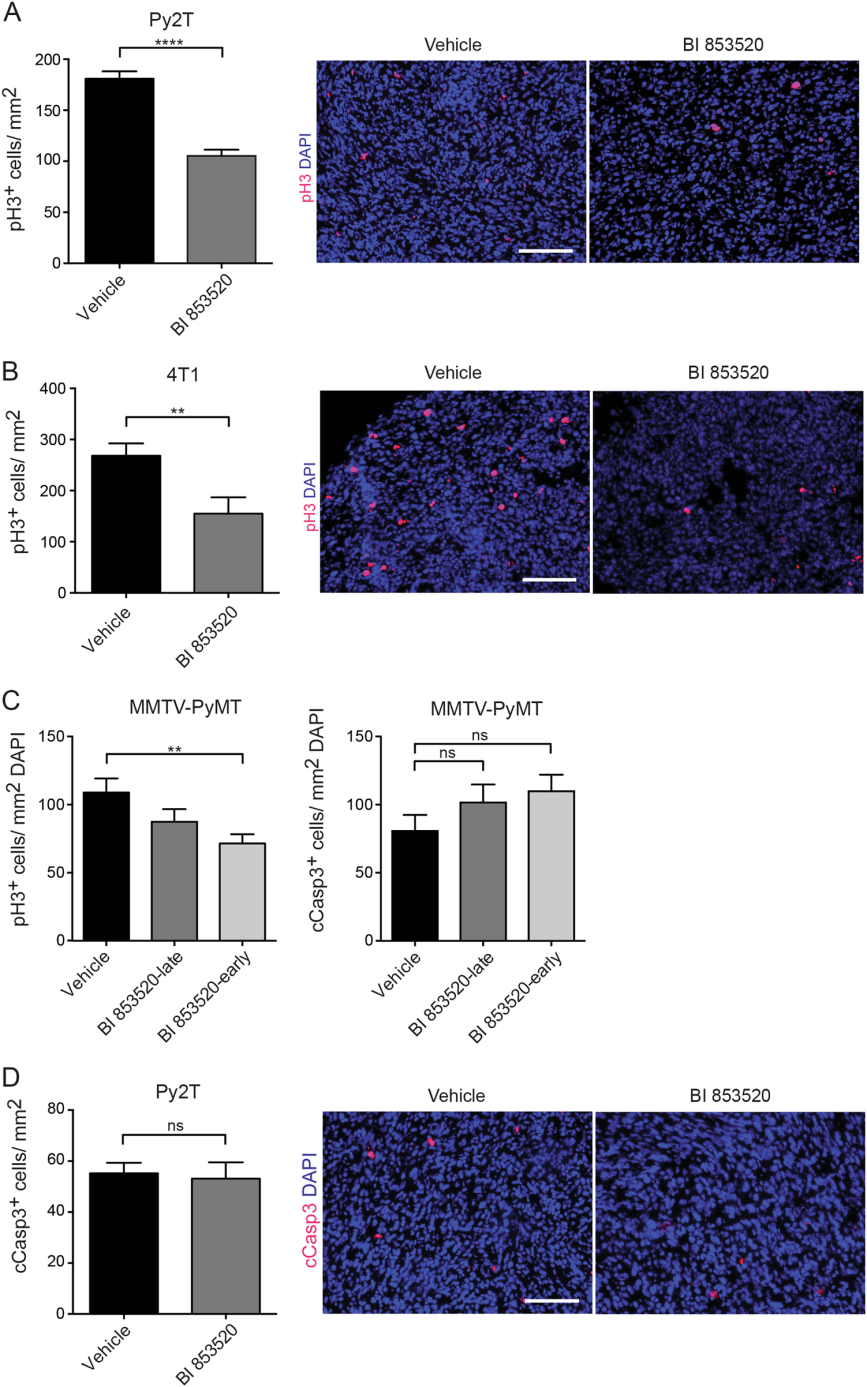
Reduced primary tumor growth is due to an anti-proliferative effect of BI 853520. **a** Immunofluorescence microscopy analysis of primary tumor sections of Py2T breast tumors of mice treated for 3 days with vehicle control or with daily 50 mg/kg BI 853520 for phospho-histone 3 (pH3; red) and DAPI (cell nuclei; blue). Quantification of pH3-positive nuclei per mm^2^ is shown on the left, representative immunofluorescence images are shown on the right. *n* = 5 tumors in the vehicle group, *n* = 3 tumors in the BI 853520-treated group. **b** Immunofluorescence microscopy analysis of primary tumor sections of 4T1 breast tumor bearing mice treated with vehicle control or daily 50 mg/kg BI 853520 and analyzed at the experimental end point as shown in Fig. 2b for pH3 (red) and DAPI (cell nuclei; blue). Quantification of pH3-positive nuclei per mm^2^ is shown on the left, representative immunofluorescence images are shown on the right. *n* = 4 tumors in the vehicle group, *n* = 3 tumors in the BI 853520-treated group. Due to the widespread necrotic areas in this tumor model, only areas with intact cell nuclei were assessed. **c** Left panel: number of pH3-positive nuclei per mm^2^ on primary tumor sections from MMTV-PyMT transgenic mice following treatment with vehicle alone, daily 50 mg/kg BI 853520-late (from 10 to 13 weeks of age) or daily 50 mg/kg BI 853520-early (from 5 to 13 weeks of age) as described in Fig. 2d. *n* = 4 tumors in the vehicle group, *n* = 5 tumors in the BI 853520-late group, and *n* = 5 tumors in the BI 853520-early group. Right panel: Number of cleaved caspase-3 (cCasp3)-positive nuclei per mm^2^ of primary tumor sections from MMTV-PyMT transgenic mice following BI 853520 treatment. *n* = 5 tumors in the vehicle group, *n* = 6 tumors in the BI 853520-late group and *n* = 7 tumors in the BI 853520-early group. Due to the massive difference in cellularity between different tumor areas in this particular tumor model, pH3, and cCasp3 counts were corrected to the area covered by DAPI-staining as a surrogate for cellularity. **d** Immunofluorescence microscopy analysis of primary tumor sections of Py2T breast tumor-bearing mice treated for 3 days with vehicle control or 50 mg/kg daily BI 853520 for cleaved caspase 3 (cCasp3; red) and DAPI (cell nuclei; blue). Quantification of cCasp3-positive nuclei per mm^2^ is shown on the left, representative immunofluorescence images are shown on the right. *n* = 5 tumors in the control group and *n* = 3 tumors in the BI 853520-treated group. Data indicate counts per field of view, shown as mean ± SEM. Statistical difference was determined by the unpaired, two-tailed Student’s *t* test. ****p < 0.0001, **p < 0.01. ns not significant

In order to validate the anti-proliferative mechanism of BI 853520 on primary tumor growth, we analyzed the proliferation marker pH3 on histological sections of tumors from mice orthotopically transplanted with Py2T cells and treated with BI 853520 for a short term. Immunostaining for pH3 demonstrated a significant reduction of pH3-positive nuclei per area in mice treated with BI 853520 (Fig. 4a). Similarly, in both the 4T1 breast cancer model and the MMTV-PyMT transgenic mouse model, BI 853520 treatment led to a significant decrease in pH3-positive nuclei per mm^2^ (Fig. 4b, c). In contrast, apoptosis as assessed by immunofluorescence staining for cleaved caspase-3 (cCasp3) was not significantly affected in the transgenic mouse model and in the short-term Py2T breast cancer model (Fig. 4c, d). In the 4T1 model, inter-experimental variability of cCasp3 staining and thus of the rate of apoptosis did not allow a reliable conclusion. Immunofluorescence staining of histological sections from the 4T1 tumors for blood vessel density (CD31), for vessel perfusion (FITC-Lectin perfusion), for vessel leakiness (FITC-Dextran), and for tumor hypoxia (Pimonidazole) did not reveal any significant changes (Suppl. Figure 5A–D).

Consistent with the findings on murine breast cancer cells cultured in vitro, these results indicate that the repressive effect of BI 853520 on tumor growth is attributable to its anti-proliferative mode of action on tumor cells.

### Heterogeneous effects of BI 853520 at different stages of the metastatic cascade

**Fig. 5:**
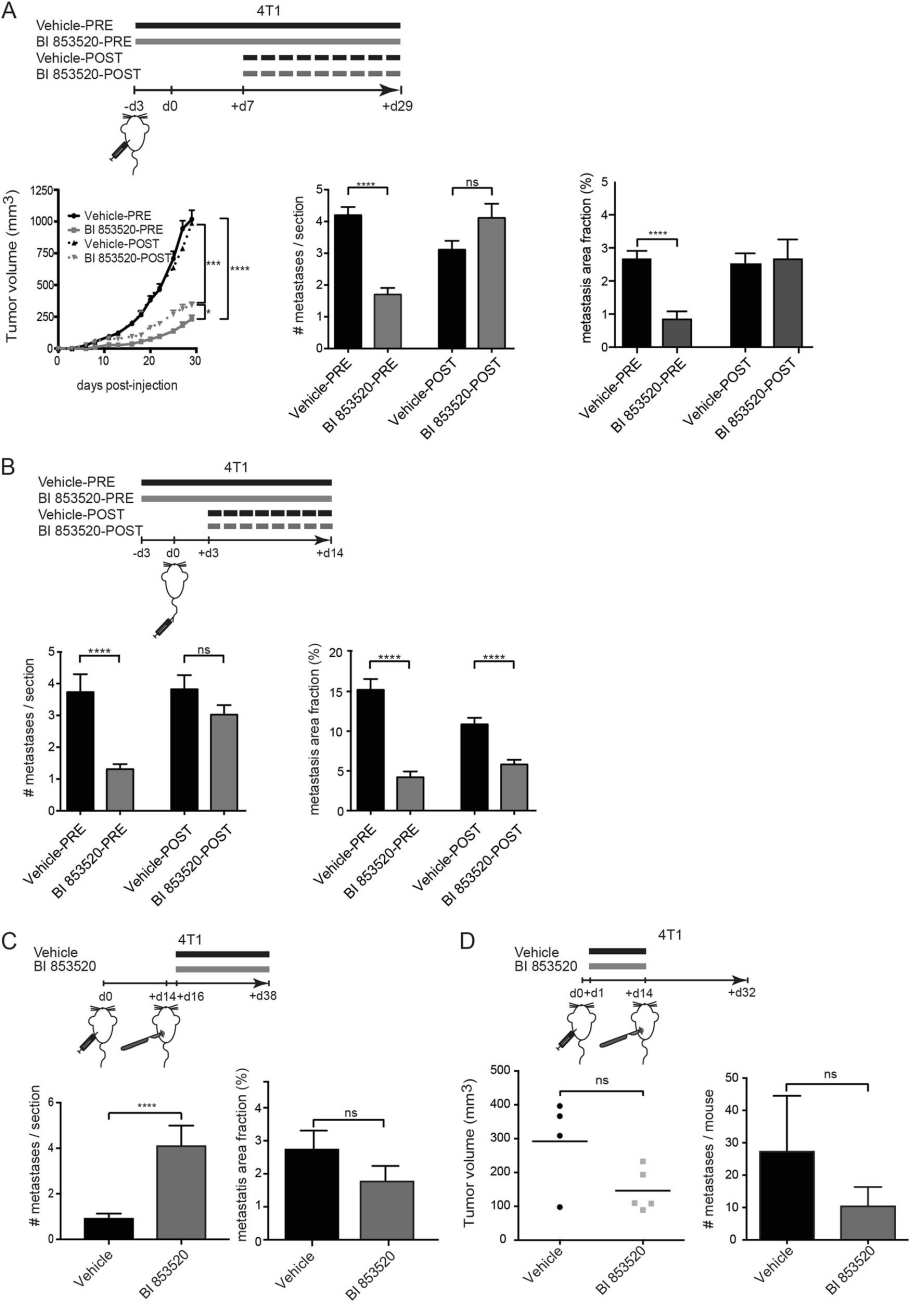
BI 853520 represses outgrowth of pulmonary metastases. **a** Top panel: Schematic overview of the experimental setup. Mice were either pre-treated with BI 853520 3 days prior to 4T1 orthotopic tumor cell inoculation (Vehicle-PRE or BI 853520-PRE groups) or treated 7 days post 4T1 orthotopic tumor cell injection (Vehicle-POST or BI 853520-POST groups). Left bottom panel: Primary tumor growth in the indicated treatment cohorts was monitored over time. BI 853520 treatment (BI 853520-PRE and BI 853520-POST) significantly reduced primary tumor growth. Middle bottom panel: Average numbers of individual pulmonary metastases per lung section. Only pre-treatment with BI 853520 decreased the numbers of pulmonary metastases. Right bottom panel: Average metastatic area fraction (%) in pulmonary cross sections. Only pre-treatment with BI 853520 decreased the outgrowth of pulmonary metastases. *n* = 9, Vehicle-PRE; *n* = 9, BI 853520-PRE; *n* = 8, Vehicle-POST; *n* = 8, BI 853520-POST. Statistical analysis was performed by unpaired, two-tailed Mann–Whitney *U* test (for tumor volumes) or unpaired, two-tailed Student’s *t* test (for number metastases/section and metastasis area fraction). **b** Top panel: Schematic overview of the experimental setup. Mice were either pre-treated with BI 853520 3 days prior intravenous injection of 4T1 cells (Vehicle-PRE or BI 853520-PRE groups) or treated 3 days post intravenous injection of 4T1 cells (Vehicle-POST or BI 853520-POST groups). Left bottom panel: Average numbers of pulmonary metastases per lung section. BI 853520 pre-treatment significantly decreased pulmonary metastases. Right bottom panel: Average metastatic area fraction (%) in pulmonary cross-sections. Both BI 853520 treatments (BI 853520-PRE and BI 853520-POST) significantly reduced the metastatic area fraction. *n* = 5 for all treatment cohorts. Statistical analysis was performed using unpaired, two-tailed Student’s *t* test. **c** Schematic overview of the experimental setup. 4T1 cells were orthotopically transplanted into the mammary fat pad, followed by primary tumor removal 14 days post-injection and BI 853520 therapy in an adjuvant setting 2 days post surgery. Mice were sacrificed 38 days post injection. Left bottom panel: Average numbers of pulmonary metastases per lung section in an adjuvant BI 853520 setting. BI 853520 significantly increased the number of pulmonary metastases. Right bottom panel: Average metastatic area fraction (%) in pulmonary cross-sections, with a trend of decrease following adjuvant BI 853520 therapy. *n* = 7, Vehicle; *n* = 5, BI 853520. Statistical analysis was performed using unpaired, two-tailed Student’s *t* test. **d** Schematic overview of the experimental setup. 4T1 cells were orthotopically transplanted into the mammary fat pad, followed by neoadjuvant BI 853520 therapy. Primary tumors were removed 14 days post injection and BI 853520 therapy was discontinued. Mice were sacrificed 32 days post injection. Left bottom panel: Primary tumor volumes. Right bottom panel: Number of metastatic lesions per mouse. *n* = 5, Vehicle; *n* = 5, BI 853520. Statistical analysis was performed by Mann–Whitney *U* test for tumor volumes and Student’s *t* test for lung metastases. ****p < 0.0001, ***p < 0.001. ns not significant

Most studies have primarily focused on primary tumor growth when validating novel anti-cancer compounds, yet metastasis is the major cause of morbidity and mortality. To this end, we performed an in-depth analysis of BI 853520's effect on the different stages of the metastatic process, employing the 4T1 transplantation model of breast cancer that predominantly metastasizes to the lung. Assuming that the metastatic process involves the interplay between cancer cells and cell types of the tumor microenvironment, we orthotopically injected 4T1 cells in BALB/c mice and started BI 853520 treatment 3 days before (BI 853520 PRE) or 7 days post 4T1 cell implantation (BI 853520 POST). Both treatment regimens efficiently reduced primary tumor volume (Fig. 5a). Notably, BI 853520 PRE-treated 4T1 tumors were significantly delayed in their initial growth, since they were palpable only at later time points as compared to the vehicle-PRE-treated control cohort (Suppl. Figure 6A). Interestingly, even though BI 853520 post treatment led to a significant decline in 4T1 tumor volumes, pulmonary metastasis numbers per section and the metastasis area fraction were only decreased in the BI 853520 PRE cohort (Fig. 5a and Suppl. Figure 6B). This result suggests that once 4T1 tumor cells have entered the lung parenchyma and proliferation has been initiated, BI 853520 treatment does no longer reduce the number of metastatic nodules. To test this hypothesis, 4T1 tumor cells were injected intravenously (i.v.) into the tail vein of mice to bypass the processes of primary tumor colonization and intravasation. BI 853520 treatment was either initiated 3 days before i.v. tumor cell injection (BI 853520 PRE) or 3 days after i.v. injection of tumor cells (BI 853520 POST)—a time point when metastatic clones have already started proliferating in the lung (Suppl. Figure 6C). Indeed, pre-treating the mice before i.v. tumor cell injection reduced the number of metastasis and the metastatic area fraction (Fig. 5b). However, initiating BI 853520 treatment after metastasis had started to form did not significantly reduce the number of metastasis but decreased metastatic nodule size, as suggested by the reduced metastatic area fraction (Fig. 5b), consistent with BI 853520's anti-proliferative activity.

The latter result would argue that BI 853520 may not be able to suppress the metastatic seeding of breast cancer cells, but rather inhibit outgrowth of already seeded metastatic modules. To test this possibility, we performed an experiment in which primary tumors were surgically resected 14 days after orthotopic implantation of 4T1 cells, and BI 853520 treatment was initiated 2 days later. Surprisingly, the number of metastatic nodules was increased; however, the metastatic area fraction was slightly reduced, yet without statistical significance (Fig. 5c and Suppl. Figure 6D). When 4T1-transplanted mice were treated with BI 853520 before surgical removal of the primary tumor and then therapy was stopped, primary tumor growth and also the number of metastasis were reduced, although without statistical significance (Fig. 5d). However, when reduced primary tumor growth was normalized to the number of metastasis (metastatic index), no significant change in the number of metastasis between BI 853520-treated and control-treated mice could be observed (Suppl. Figure 6E).

A similar overall lack of significant suppression of metastasis by BI 853520 was also seen in the MMTV-PyMT mouse model of metastatic breast cancer. Although primary tumor growth and tumor progression was strongly reduced (Fig. 2d), the number of metastases was unchanged regardless of initiating BI 853520 treatment at an early or a late stage of multi-step carcinogenesis (Suppl. Figure 6F). However, comparable to the 4T1 highly metastatic, syngeneic transplantation model of breast cancer, in the MMTV-PyMT transgenic mouse model of stepwise breast cancer progression and metastasis, the percentage of large metastases was significantly reduced by long-term BI 853520 treatment (Suppl. Figure 6F), suggesting that BI 853520 repressed metastatic tumor cell proliferation. Together, these results indicate that BI 853520 rather represses tumor cell proliferation than metastatic dissemination and that BI 853520 seems to exert heterogeneous effects on metastasis formation depending on the time and the stage of the metastatic cascade when BI 853520 is first administered.

### E-cadherin status-dependent effects by BI 853520

**Fig. 6:**
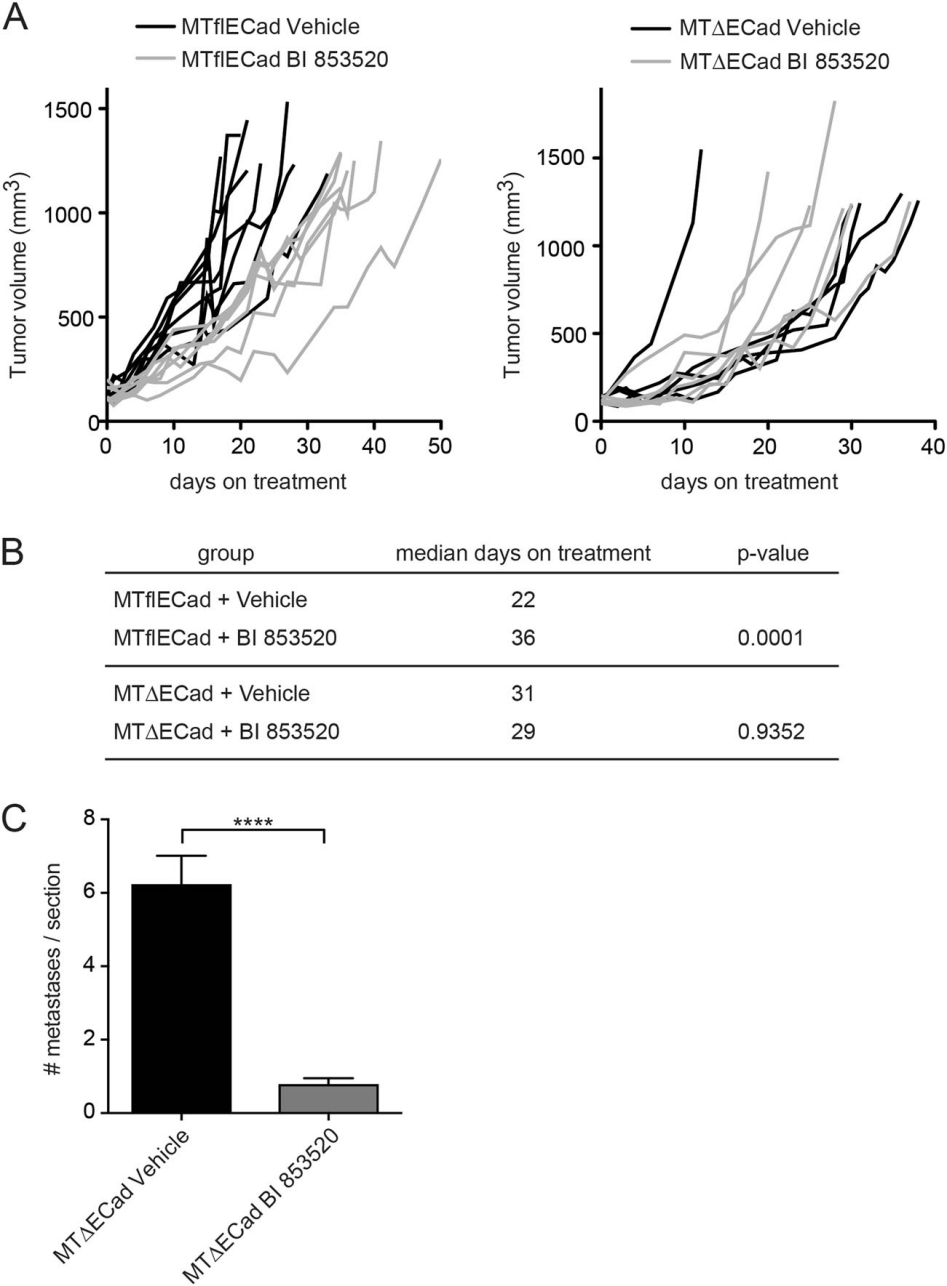
E-cadherin-dependent repression of metastasis formation by BI 853520. **a** Mice were orthotopically transplanted with E-cadherin-expressing MTflECad or with E-cadherin-deficient MTΔECad murine breast cancer cells. BI 853520 treatment was started at a primary tumor size of 100 mm^3^, and animals were sacrificed before the primary tumor reached a size of ~1500 mm^3^. BI 853520 significantly increased survival of mice transplanted with MTflECad cells, but not in mice transplanted with MTΔECad cells. **b** BI 853520 significantly increases the mean time to reaching termination criteria in mice transplanted with E-cadherin-proficient MTflECad cells, but not in mice transplanted with E-cadherin-deficient MTΔECad cells. Statistical analysis was performed using a log-rank (Mantel–Cox) test. MTflECad: vehicle cohort, *n* = 8; BI 853520 cohort, *n* = 7. MTΔECad: vehicle cohort, *n* = 5; BI 853520 cohort, *n* = 6. **c** BI 853520 reduces the average numbers of pulmonary metastases per lung section in the cohort of mice bearing MTΔECad tumors. Vehicle cohort, *n* = 5; BI 853520 cohort, *n* = 6. Statistical analysis was performed using unpaired, two-tailed Student’s *t* test. All data are depicted as mean ± SEM. ****p < 0.0001

FAK is seen as a key mediator in the crosstalk of integrin-mediated cell–matrix and E-cadherin-mediated cell–cell contacts—central mechanisms involved in cancer cell invasion and metastasis^32^. In order to study the influence of the E-cadherin status and, with it, of an EMT on the cells’ susceptibility to BI 853520, we employed the E-cadherin-proficient and E-cadherin-deficient murine breast cancer cell lines MTflECad and MTΔECad, respectively. MTflECad cells exhibit a fully differentiated epithelial cell phenotype, while the genetic ablation of E-cadherin expression in MTΔECad resulted into a complete EMT, as manifested by changes in marker gene expression and a gain in the cells’ migratory, invasive, and metastatic capabilities^29^. MTflECad and MTΔECad cells were orthotopically injected into immunodeficient Rag2^−/^^−^;γc^−/−^ mice, and BI 853520 treatment was initiated when tumors reached the size of 100 mm^3^ and continued until tumors measured ~1500 mm^3^. Strikingly, BI 853520 treatment significantly delayed primary tumor growth of the highly differentiated, E-cadherin-proficient and non-metastatic MTflECad tumors (Fig. 6a, b), as already described above (Fig. 2c). In contrast, the growth of E-cadherin-deficient, invasive and metastatic MTΔECad tumors was not apparently affected by treatment with BI 853520 (Fig. 6a, b). Notably, BI 853520 reduced the number of metastasis in the lungs of mice transplanted with MTΔECad cells (Fig. 6c), whereas MTflECad did not form metastasis even in the absence of BI 853520 (data not shown).

In summary, the data suggest that BI 853520 is more potent in repressing tumor cell proliferation and primary tumor growth of differentiated, E-cadherin-expressing tumor cells (MTflECad, Py2T, in parts 4T1 cells and early stages of MMTV-PyMT tumor progression), while it represses the metastatic outgrowth of invasive, E-cadherin-deficient tumor cells (MTΔECad and late stages/metastasis of MMTV-PyMT tumor cells). The loss of E-cadherin expression is a hallmark of an EMT. While an EMT is mainly responsible for primary tumor cell invasion, its reversal, a mesenchymal–epithelial transition (MET), has been shown to contribute to the metastatic outgrowth of disseminated cancer cells in distant organs. Hence, the repressive effect of BI 853520 on metastatic outgrowth may be a result of the more potent anti-proliferative effect of BI 853520 on differentiated cancer cells.

## Discussion

A plethora of reports have highlighted that FAK’s expression levels and its activation correlate with the initiation, progression, and the prognosis of a wide variety of malignancies, and, thus, FAK has been repeatedly proposed as an attractive target for cancer therapy^2–4,6,7,20^. Subsequently, a number of laboratories and pharmaceutical companies have invested into the development of specific FAK-Is that are now evaluated in pre-clinical animal models and in first clinical trials^6,20–22^. However, the actual mode of action of FAK in tumor progression and metastasis and the biological consequences of its pharmacological inhibition are still poorly understood.

Here, we have used the novel and highly specific small-molecule FAK-I BI 853520^24^ to repress FAK activity in cultured breast cancer cells *in vitro* and in various preclinical mouse models of breast cancer (Py2T, 4T1, MTflECad/MTΔECad, and MMTV-PyMT) in vivo. BI 853520 elicits a substantial repression of cancer cell proliferation and thus a repression of primary tumor growth and outgrowth of metastatic lesions. However, BI 853520 does not diminish the actual metastatic dissemination of murine breast cancer cells, in contrast we observe an increased number of pulmonary metastases upon treatment with BI 853520 in an adjuvant setting after surgical resection of 4T1 primary tumors.

On the other hand, BI 853520 most effectively hampers the establishment of pulmonary metastasis in mice harboring E-cadherin-deficient (MTΔECad) tumors (Fig. 6c), the metastatic outgrowth of 4T1 breast cancer cells with downregulated E-cadherin expression (Fig. 2a), and the 3D Matrigel invasion of mesenchymal Py2T-LT cells (Fig. 1f and Suppl. Figure 2). Shapiro et al.^33^ have linked FAK-I susceptibility of mesothelioma growth to merlin deficiency. However, the Py2T, MTflECad, and MTΔECad cells employed here express similar levels of merlin, and the susceptibility of Py2T and MTflECad tumors and the insensitivity of MTΔECad tumors to BI 853520 appears to be independent of merlin expression status (data not shown). Rather, our results suggest that the lack or low expression of E-cadherin in 4T1 and MTΔECad cells and in later stages of MMTV-PyMT tumor progression, and thus the invasive nature of the cancer cells, may serve as a molecular biomarker for an anti-metastatic activity of BI 853520. Intriguingly, this notion stands in stark contrast to previous studies, where FAK inhibition activates E-cadherin function and the reversal of an EMT, thereby restraining the initiation and establishment of proliferative programs by micrometastatic cell clusters and not affecting macrometastatic expansion^34–39^. However, our histopathological analysis and gene expression profiling of primary tumors treated or not with BI 853520 have not revealed marked changes in the EMT status of the breast cancer cells. Hence, further investigations are warranted to decipher whether E-cadherin expression can be used as a potential predictive marker for the subset of patients that will respond to anti-metastatic FAK-I therapy^3,18,20,21^. Notably, Hirt et al.^24^ have recently reported that the expression of hsa-miR-200c-3p, an epithelial-specific microRNA efficiently repressing EMT transcription factors and thus promoting E-cadherin expression, strongly correlated with BI 853520 therapeutic efficacy in a number of transplantation mouse models of various cancer types. Confronted with these seemingly conflicting results, we hypothesize that intermediate stages of an EMT, such as a partial EMT with a combination of epithelial and mesenchymal marker expression, may represent a cell status of high cell plasticity and metastasis and thus may be preferentially targeted by FAK inhibition.

We have subsequently explored the direct biological effects of FAK inhibition on tumor cells and characterized potential mechanisms responsible for the significant decrease in tumor burden following BI 853520 administration. RNA sequencing of primary murine breast tumors treated with BI 853520 reveals a potent repression of tumor cell proliferation and thus primary tumor growth. Gene set enrichment analysis confirms a significant reduction in the relative expression of genes important for a positive regulation of mitotic cell cycle (including Cdk1 and Cdk4) and an increase in the expression of genes relevant for a repression of the cell cycle. Previous studies have indicated that ectopic expression of a constitutive-active form of FAK leads to a rapid induction of proliferation in murine mammary tumor cells, whereas a decrease in FAK expression impairs their cell proliferation in vitro and in vivo^6,10,17,40^. In line with these findings, BI 853520 treatment elicits a significant reduction in murine breast cancer cell proliferation in vitro and in vivo. These findings are supported by flow cytometry-based cell cycle analysis where increasing concentrations of BI 853520 up to 1 µM shift cells from the S phase to the G1 phase. Together, the data suggest a linear mechanistic relationship between FAK activity and murine breast cancer cell proliferation.

Previously, it has been reported that tumor cells, which have been genetically ablated for FAK expression or treated with various FAK-I, undergo cell death^6,17,20,41^. However, using immunofluorescence analysis of primary tumor sections and cellular assays, we have not observed a significant induction of apoptosis following BI 853520 treatment. We conclude that the most prominent therapeutic effect observed by BI 853520 is based on its anti-proliferative activities. Along these lines, the results reported here also indicate that the kinase activity of FAK is required for 4T1 murine breast cancer cell pulmonary metastasis: the pharmacological inhibition of FAK kinase activity by BI 853520 is consistent with the previously reported findings that the kinase domain of FAK is required for metastatic outgrowth of 4T1 cells^42^.

Although ongoing clinical trials with various FAK-I seem encouraging and methods for patient stratification are planned, it is questionable whether FAK inhibition will be used as a single-agent therapy, as it is the case for most targeted therapies^13,15,20^. Previous studies have shown that repression of FAK signaling in combination with diverse chemotherapies enhanced chemo-cytotoxicity in vitro and in vivo^43–53^. In line with FAK's role in an anti-tumor evasion mechanism and an upregulated immune response following BI 853520 treatment in the 4T1 breast cancer model (Fig. 3b), it has been shown in mouse models of squamous cell cancer and pancreatic ductal adenocarcinoma that FAK inhibition increases immune surveillance, enhances the sensitivity to T cell immunotherapy and treatment with PD-1 antagonists, and ultimately delays tumor progression^54–56^. Future efforts should be made to test BI 853520 and other FAK-I in combination with chemotherapy or immunotherapy reagents, thereby evaluating not only the anti-proliferative effects but also the anti-metastatic effects of a combination therapy that might be applicable for various cancer types^3,22,41,57^.

## Materials and methods

### Treatment of tumor transplantation and transgenic mouse models

The murine metastatic cell line 4T1 is described elsewhere^28,58^. Py2T cells have been derived from a breast tumor of an MMTV-PyMT female mouse with an FVB/N background^25^. E-cadherin-proficient MTflECad cells have been isolated from a lymph node metastasis of a MMTV-Neu;*Cdh1*fl/fl tumor-bearing mouse and have been infected with an adenovirus-expressing Cre recombinase to obtain the E-cadherin-deficient cell line MTΔECad^29^. All cell lines were maintained in Dulbecco’s modified Eagle's medium (DMEM), supplemented with fetal calf serum (10%), glutamine (2 mM), penicillin (100 U; Sigma-Aldrich), and streptomycin (0.2 mg/l; Sigma-Aldrich) in a humidified atmosphere of 37 °C, 5% CO_2_, and 95% humidity.

Py2T, MTflECad, or MTΔECad cells (0.5 × 10^6^) or 4T1 cells (1 × 10^6^) suspended in 100 µl sterile phosphate-buffered saline (PBS) were injected into mammary fat pad number nine of anesthetized, 6–10-week-old female FVB/N (Py2T cells), BALB/c (4T1 cells), Rag2^−/^^−^;γc^−/−^, or nude mice (BALB/cAnN-*Foxn1nu/nu*/Rj; Janvier) mice (MTflECad and MTΔECad cells). The length (*D*) and width (*d*) of the tumors was measured by a digital caliper every 2 to 3 days and the tumor volume was calculated according the formula (*V* = 0.543 × *D* x *d*^2^). Treatment protocols were initiated several days after tumor cell inoculation; the details are indicated in the respective figure legends. BI 853520^24^ was dissolved in 0.5% hydroxyethylcellulose natrosol and administered at 50 mg/kg body weight daily by oral gavage.

The MMTV-PyMT transgenic mouse model of metastatic breast cancer was used to determine the therapeutic and biological effect of BI 853520^27,59,60^. BI 853520 was administered orally each day to mice of 5 weeks of age for 8 consecutive weeks, referred to as “early BI 853520” treatment. In another cohort of mice, BI 853520 was administered orally each day to mice of 10 weeks of age for three consecutive weeks, referred to as “late BI 853520” treatment. All mice of this experimental setup were sacrificed at 13 weeks of age and primary tumors and lungs were used for further processing.

For RNA sequencing, BI 853520 was administered from day 15 post injection for five consecutive days. To determine whether BI 853520 pre-treatment is able to prime the tumor microenvironment, mice were either pre-treated 3 days prior tumor cell inoculation (Vehicle PRE or BI 853520 PRE group) or BI 853520 was administered 7 days (subcutaneous tumor cell injection) or 3 days (i.v. tumor cell injection) post tumor cell injection (Vehicle POST or BI 853520 POST group).

For i.v. injection, 0.5 × 10^6^ mouse 4T1 breast cancer cells were injected into the tail vein of BALB/c mice.

To mimic the patient situation, BI 853520 was administered in an adjuvant setting to the preclinical 4T1 breast cancer mouse model. For this purpose, primary tumors were surgically removed 14 days after implantation of 4T1 cells in to the mammary fat pad, and BI 853520 therapy was started 2 days post surgery for the consecutive 22 days.

For neoadjuvant BI 853520 treatment, BI 853520 administration was initiated 1 day after orthotopic tumor cell implantation (1 × 10^6^ 4T1 cells mixed with Matrigel) until primary tumor removal on days 14 or 15 post tumor cell inoculation. Lung metastases were analyzed 32 days post tumor cell inoculation.

When not specified otherwise, animals were sacrificed when the maximum allowed primary tumor size of 1.5 cm^3^ was reached. For all experiments, primary tumors, lungs, and peripheral blood were isolated and used for further processing as previously described^25,61^. Primary tumors in the abdominal mammary fat pad of MMTV-PyMT mice were graded by using a Zeiss Axio Imager.Z2 microscope and ZEN 2 Zeiss Microscopy software (version 2.0). Pulmonary cross-sections were imaged with a ×10 objective of a Zeiss Axio Imager.Z2 or a Zeiss Axio Observer (Zeiss) microscope and the average amount of unique pulmonary metastases as well as the average metastatic area fraction (%) was quantified by using the ImageJ software (ImageJ, Wayne Rasband, National Institutes of Health, USA), ZEN 2 Zeiss Microscopy software, or a customized application from Visiopharm. All animal experiments were approved by and performed according to the guidelines and legislation of the Swiss Federal Veterinary Office and the Cantonal Veterinary Office, Basel-Stadt, Switzerland (permit numbers 1878, 1907, and 1908).

### BI 853520 treatment of breast cancer cells in vitro

For immunoblotting analysis, immunofluorescence staining (for phospho-H3 and cCasp3) and EdU/PI cell cycle analysis, 1.3 × 10^5^ 4T1 cells were plated in a 6-cm dish. For immunofluorescence staining (for phospho-Y397-FAK and total FAK), 3.3 × 10^4^ 4T1 cells were plated in a 6-cm dish. For cell viability determination following 96 h treatment with BI 853520, 0.5 × 10^3^ 4T1, Py2T, and Py2T-LT cells were seeded in a 96-well format. 4T1, Py2T, and Py2T-LT cells were treated for the indicated time points with varying concentrations of BI 853520.

### Immunoblotting analysis

Tumor pieces were snap frozen and lysates were used for immunoblotting analysis. Snap-frozen Py2T primary tumor pieces were homogenized by a Polytron PT1200 E (Kinematica AG) in lysis buffer (50 mM HEPES, pH 7.5, 150 mM NaCl, 1% glycerol, 1% Triton X-100, 1.5 mM MgCl_2_, 5 mM EGTA) supplemented with 0.1 mM sodium orthovanadate (Sigma-Aldrich), 20 mM sodium fluoride (Sigma-Aldrich), and a protease inhibitor cocktail (Sigma-Aldrich, 1:200). The lysates were cleared by centrifugation and the protein content of supernatants was determined by using Protein Assay Dye Reagent Concentrate (Bio-Rad). Snap-frozen 4T1 primary tumor pieces were homogenized by metal bead lysing matrix (MP Biomedicals) in RIPA buffer (Sigma-Aldrich, 150 mM NaCl, 1.0% IGEPAL^®^ CA-630, 0.5% sodium deoxycholate, 0.1% sodium dodecyl sulfate (SDS), 50 mM Tris, pH 8.0) supplemented with 20 mM sodium fluoride (Sigma-Aldrich) and a protease inhibitor cocktail (Sigma-Aldrich, 1:100). 4T1, Py2T, and Py2T-LT cells previously being treated with BI 853520 were lysed in a sample solution (0.5 M Tris-HCl, pH 6.8, 10% SDS, glycerol) or an NP40 buffer (20 mM Tris, pH 7.5, 100 mM NaCl, 5 mM MgCl_2_, 0.2% NP40, 10% glycerol, 1 mM NaF, 20 mM β-glycerophosphate, and freshly added 0.5 mM dithiothreitol/1× PIC (Protease Inhibitor Cocktail) (Sigma)/1 mM vanadate). The protein content was determined by the Pierce BCA Protein Assay Kit (Thermo Scientific), loaded on 10% SDS-polyacrylamide gel electrophoresis gels, and subsequently used for immunoblotting analysis. Following blocking of the nitrocellulose blotting membranes (GE Healthcare) for 1 h in 5% bovine serum albumin (BSA; Sigma-Aldrich) dissolved in 0.1% Tween (Sigma-Aldrich) in TBS (for Py2T tumors) or PBS (for 4T1 tumors), membranes were incubated with primary antibodies: mouse anti-FAK (Millipore, 05-537, clone 4.47), rabbit anti-pFAK (Y397, Invitrogen, 44-624G), rabbit anti-PYK2 (Abcam, ab32571, clone YE353), and rabbit anti-pPYK2 (Y402, Abcam, ab4800) overnight at 4 °C. GAPDH (glyceraldehyde 3-phosphate dehydrogenase) (Abcam, ab9485 or Sigma-Aldrich, G8795) or α-tubulin (Sigma-Aldrich, T-9026, clone DM1A) were used as a loading control. Membranes were then incubated for 1 h with horseradish peroxidase-conjugated secondary antibodies (Jackson) and subsequently visualized using Fusion Fx chemoluminescence reader (Vilber Lourmat) and Curix 60 (AGFA). For Py2T tumors, following visualization of the pFAK signal, antibodies were removed by using a stripping buffer (0.2 M glycin, pH 2.5, 0.1% SDS, 1% Tween) two times 30 min before initiating the staining procedure against total FAK protein.

### Immunofluorescence staining of frozen sections

To prepare cryostat sections, organs were fixed for 2 h at 4 °C in 4% paraformaldehyde, incubated overnight in 20% sucrose in PBS (Sigma-Aldrich) at 4 °C, and then embedded and snap frozen in OCT freezing matrix (Tissue Tek). For immunofluorescence analysis of frozen sections, cryosections (7 µm thick) of tumor samples were dried for 30 min and rehydrated three times for 5 min in PBS. After permeabilization for 20 min with 0.2% Triton X-100 (Sigma-Aldrich) in PBS, slides were washed three times for 5 min in PBS, blocked for 1 h at room temperature in 5% goat serum diluted in PBS (blocking buffer) for the phospho-H3 and CD31 staining and 20% goat serum diluted in PBS for the cCasp3 staining, and incubated overnight at 4 °C with primary antibodies (rabbit anti-mouse phospho-H3, Millipore, 06-570; rat anti-mouse CD31, BD Pharmingen, 550274; rabbit anti-mouse cCasp3, Cell Signaling, 9664, clone 5A1E) diluted in blocking buffer.

To detect perfused vessels, 150 µg of fluorescein-labeled *Lycopersicon esculentum* lectin (Vector Laboratories, GL-1171) was injected i.v. and allowed to circulate for 20 min. Next, terminally anesthesized animals were perfused with 4% paraformaldehyde (PFA) in PBS followed by PBS via the left cardiac ventricle.

For the detection of leaky blood vessels, 250 μg fluorescein-labeled dextran (70 kDa; Life Technologies, D-1822) was injected i.v. in a tail vein and allowed to circulate for 5 min. Then, terminally anesthesized animals were perfused with PBS followed by a perfusion with 4% PFA in PBS via the left cardiac ventricle.

Hypoxic areas were detected by injecting pimonidazole-HCl (Hypoxyprobe Omni Kits, Hypoxyprobe, Inc.) at 60 mg/kg intraperitoneally 2 h prior to sacrifice of the animals.

Immunofluorescence stainings were revealed by incubating slides for 1 h at room temperature with Alexa Fluor 488-labeled, Alexa Fluor 568-labeled, Alexa Fluor 633-labeled, or Alexa Fluor 647-labeled secondary antibodies (Invitrogen, 1:200) diluted in blocking buffer and washed three times for 5 min in PBS. Nuclei were counterstained for 10 min with 4′,6-diamidino-2-phenylindole (DAPI, Sigma-Aldrich, 1:5000) and slides were washed three times for 5 min in PBS. Finally, slides were mounted in DAKO fluorescence mounting medium and evaluated with a ×20 objective on a DMI 4000 microscope (Leica). Images were processed and analyzed using the ImageJ software (ImageJ, Wayne Rasband, National Institutes of Health, USA).

### Immunofluorescence staining of cultured cells

4T1 cells grown on coverslips were fixed for 20 min with 4% PFA in PBS at room temperature. Subsequently, cells were permeabilized for 10 min with 0.1% Triton X-100 (Sigma-Aldrich) in PBS and blocked with 1% BSA (Sigma-Aldrich) in PBS for 2 h. To visualize FAK and pFAK expression, cells were incubated with the primary antibodies mouse anti-FAK (Millipore, 05-537, clone 4.47), rabbit anti-pFAK (Y397, Invitrogen, 44-624G), rabbit anti-mouse phospho-H3 (Millipore, 06-570), rabbit anti-mouse cCasp3 (Cell Signaling, 9664, clone 5A1E) diluted in 1% BSA in PBS overnight at 4 °C, followed by a 30-min incubation with DAPI (Sigma-Aldrich, 1:5000) and Alexa Fluor 488-labeled or Alexa Fluor 568-labeled secondary antibodies (Invitrogen, 1:200). The cells were sealed by mounting them with Dako fluorescence mounting medium. Images were obtained using a Confocal SP5 microscope with a ×63 objective and a DFC 360 FX Monochromatic Camera (Leica) or with a ×10 (cCasp3) or ×20 objective (pH3) on a DMI 4000 microscope (Leica).

### In-gel immunofluorescence staining

Growth factor-reduced Matrigel (Corning) was diluted with ice-cold, serum-free growth medium (F12-HAM, Sigma-Aldrich) to 4 mg/ml protein concentration by gentle pipetting. Pellets of 1.5 × 10^3^ 4T1 cells and 2.5 × 10^3^ Py2T and Py2T-LT cells, respectively, were resuspended in 10 µl 4 mg/ml Matrigel and seeded into each inner well of a µ-slide angiogenesis microscopy slide (ibidi, Martinsried, Germany). Following 20 min of gel solidification in a humidified atmosphere at 37 °C and 5% CO_2_, 30 µl of normal growth medium (supplemented or not with 2 ng/ml TGFβ) was added to each well. Growth medium was refreshed every third day. After 5 days of culture, medium containing increasing concentrations of BI 853520 (0.01 µM, 0.1 µM, and 1 µM) was added. The next day, structures were fixed in the matrix for 10 min with 4% PFA in PBS, washed for 5 min with 20 mM glycine/PBS, and permeabilized and blocked for 2 h at room temperature in immunofluorescence (IF) buffer (0.2% Triton X-100/0.1% BSA/0.05% Tween-20/PBS) supplemented with 10% goat serum. Samples were stained with primary antibodies in IF buffer (rabbit anti-pFAK (Y397, Invitrogen, 44-624G); rabbit anti-ZO-1 (Zymed, 617300); mouse anti-vimentin (Sigma-Aldrich, V2258)) for 2 h at room temperature, washed twice with IF buffer, and incubated with Alexa Fluor 488-labeled or Alexa Fluor 568-labeled secondary antibodies diluted in IF buffer (Invitrogen, 1:200) for 45 min. Nuclei were stained using DAPI (Sigma-Aldrich, 1:5000) for 20 min at room temperature. Following two washes with IF buffer, samples were mounted with DAKO fluorescent mounting medium and imaged using a confocal microscope (SP5 confocal microscope with a ×63 objective, Leica).

### Cell proliferation assay

The effects of varying BI 853520 concentrations on cell proliferation were determined by an MTS assay^26^ (Promega) according to the manufacturer’s protocol. Cells were seeded, and 1 day later, varying concentrations of BI 853520 were added and cell viability was measured on day 5. Cell viability was measured using a SpectraMax 340 Microplate Reader (Molecular Devices) with a filter of 492 nm. For analysis, background absorbance from a “no-cell” control row was subtracted from all measured values and cell number of BI 853520-treated cells was normalized to the dimethyl sulfoxide-treated controls.

### Cell cycle analysis

For Edu/PI incorporation cell cycle analysis, 4T1 cells were treated for 24 h with varying concentrations of BI 853520, and the cells were incubated for 5 min with 10 µM EdU (EdU Flow Cytometry Kit, baseclick). Harvested cells were subsequently fixed for 15 min with 4% PFA in PBS at room temperature and permeabilized for 20 min using 0.5% Triton X-100 (Sigma-Aldrich) in PBS at room temperature. Following a 30 min incubation with the click reaction containing the catalyst solution, the dye azide, buffer additive, and PBS, cells were stained with PI (Sigma-Aldrich) for 2 h at a humidified atmosphere of 37 °C and 5% CO_2_. Cell cycle distribution was analyzed by using a BD FACSCANTO II analyzer and BD FACSDIVA software (BD Biosciences).

### Soft agar colony formation assay

A bottom layer of 1.5 ml 0.5% agarose/DMEM complete growth medium per 6-well plate with 2 ng/ml TGFβ and varying concentrations of BI 853520 was allowed to solidify for 30 min at room temperature. Three milliliters of 0.3% agarose/DMEM complete growth medium with 2 × 10^4^ Py2T-LT cells, 2 ng/ml TGFβ, and the target BI 853520 concentration were added on top and incubated for 1.5 h in a tissue culture incubator. Finally, 1 ml DMEM complete growth medium with 2 ng/ml TGFβ and the target BI 853520 concentration was added on top. Every 4–6 days, 0.5 ml DMEM complete growth medium supplemented with 2 ng/ml TGFβ and the target BI 853520 concentration was refreshed. After 18–21 days, colonies were stained with MTT (3-(4,5-dimethyl-2-thiazolyl)-2,5-diphenyl-2*H*-tetrazolium bromide) solution (Sigma-Aldrich) and quantified.

### Apoptosis assay

Py2T and Py2T-LT cells (2 × 10^5^) (constantly cultured in the presence of 2 ng/ml TGFβ) were plated per 60 mm petri dish and allowed to adhere overnight. The next day, BI 853520 at different concentrations was added. After 24 h of incubation with BI 853520, cells were trypsinized, stained with Cy5 Annexin-V (BD Biosciences), and analyzed by flow cytometry on a BD FACSCANTO II analyzer and BD FACSDIVA software (BD Biosciences). Apoptotic cells were defined as Annexin-V positive.

### Matrigel invasion assay

Growth factor-reduced Matrigel (Corning) was diluted with ice-cold serum-free DMEM (Sigma-Aldrich) by gentle pipetting using pre-chilled pipette tips to 4 mg/ml protein (100%) concentration. Ten microliters of Matrigel was plated into each inner well of a µ-slide angiogenesis microscopy slide (ibidi, Martinsried, Germany). The gel was allowed to solidify for 45 min in a humidified atmosphere at 37 °C and 5% CO_2_. 4T1 cells (0.3 × 10^3^) and Py2T (1.25 × 10^3^) epithelial murine breast cancer cells and Py2T-LT mesenchymal cells, respectively, were seeded on the gel, and then a top layer of growth medium with 10% Matrigel, in the case for Py2T and Py2T-LT supplemented with twice the final concentration of BI 853520 and 4 ng/ml TGFβ (for Py2T-LT), was added. Following 1 day of incubation in a humidified atmosphere at 37 °C and 5% CO_2_, twice the final concentration of BI 853520 was also added to the 4T1 cells. Medium containing 5% Matrigel and BI 853520, and in the case of Py2T-LT cells TGFβ, was replaced after 3 days, and after 6 days pictures were taken using a Leica DMIL microscope.

### RNA isolation, RNA-sequencing library preparation, sequencing, and data analysis

Snap-frozen mouse tumor pieces were homogenized in Trizol (Sigma-Aldrich) and total RNA was isolated with the RNeasy Lipid Tissue Mini Kit (Qiagen). Approximately 80 ng of total RNA was subsequently used for TruSeq Stranded mRNA Library preparation on the NeoPrep System (Illumina, San Diego, CA, USA) and sequenced on the HiSeq1500 with the paired-end 50 cycle protocol and the fast-output mode (Illumina, San Diego, CA, USA). The RNA sequencing data are available under GEO accession number GSE116117.

Paired-end RNA-sequencing (RNA-seq) reads were mapped to the mouse genome assembly, version mm9, with RNA-STAR^62^, with default parameters except that only unique hits were aligned to the genome (outFilterMultimapNmax = 1) and that reads without evidence in spliced junction table were filtered (outFilterType = “BySJout”). Using RefSeq mRNA coordinates from UCSC (genome.ucsc.edu, downloaded in July 2013) and the qCount function from QuasR package (version 3.12.1)^63^, gene expression was quantified as the number of reads that started within any annotated exon of a gene. The differentially expressed genes were identified using the edgeR package^64^. Genes with a *p* value smaller than 0.05 and a minimum log 2 fold change of ±0.584 were considered statistically significant and these genes were subjected to downstream functional analysis.

### Functional enrichment analysis

Functional enrichment analysis of differentially expressed genes for biological processes^65^ or pathways was performed in R using several publically available Bioconductor resources including GO.db (version 3.4.1), GOstats (version 2.42.0)^66^, KEGG.db (version 3.2.3), and ReactomePA (version 1.20.2)^67^. The significance of each biological processes or pathways identified was calculated using the hypergeometric test (equivalent to Fisher’s exact test). *p* Values ≤0.05 were considered statistically significant. Clustering and heatmaps of the enriched biological processes were generated using hierarchical clustering and distances between the clusters were computed using the average linkage method.

### Statistical analysis

Graphics and statistical analysis were performed using the GraphPad Prism Software Version 7.0. When not specified otherwise, the unpaired, two-tailed Student’s *t* test or Mann–Whitney *U* test were applied (****p < 0.0001; ***p < 0.001; **p < 0.01; *p < 0.05) to determine statistical significance. Quantitative data were depicted as means ± standard error of the mean (SEM).

## Electronic supplementary material

Supplementary Material

## References

[1] Acharyya S (2012). A CXCL1 paracrine network links cancer chemoresistance and metastasis. Cell.

[2] Dunn KB, Heffler M, Golubovskaya V (2010). Evolving therapies and FAK inhibitors for the treatment of cancer. Anticancer Agents Med. Chem..

[3] Lee BY, Timpson P, Horvath LG, Daly RJ (2015). FAK signaling in human cancer as a target for therapeutics. Pharmacol. Ther..

[4] Golubovskaya V (2010). Focal adhesion kinase as a cancer therapy target. Anticancer Agents Med. Chem..

[5] Schaller MD (2010). Cellular functions of FAK kinases: insight into molecular mechanisms and novel functions. J. Cell. Sci..

[6] Yoon H, Dehart JP, Murphy JM, Lim STS (2015). Understanding the Roles of FAK in Cancer: inhibitors, genetic models, and new insights. J. Histochem. Cytochem..

[7] Hao H (2009). Focal adhesion kinase as potential target for cancer therapy. Oncol. Rep..

[8] Infusino GA, Jacobson JR (2012). Endothelial FAK as a therapeutic target in disease. Microvasc. Res..

[9] Lim Y (2008). PyK2 and FAK connections to p190Rho guanine nucleotide exchange factor regulate RhoA activity, focal adhesion formation, and cell motility. J. Cell Biol..

[10] Provenzano PP, Keely PJ (2009). The role of focal adhesion kinase in tumor initiation and progression. Cell Adhes. Migr..

[11] Kleinschmidt EG, Schlaepfer DD (2017). Focal adhesion kinase signaling in unexpected places. Curr. Opin. Cell Biol..

[12] Brami-Cherrier K (2014). FAK dimerization controls its kinase-dependent functions at focal adhesions. EMBO J..

[13] Zhao X, Guan JL (2011). Focal adhesion kinase and its signaling pathways in cell migration and angiogenesis. Adv. Drug Deliv. Rev..

[14] Cai X (2008). Spatial and temporal regulation of focal adhesion kinase activity in living cells. Mol. Cell. Biol..

[15] Lechertier T, Hodivala-Dilke K (2012). Focal adhesion kinase and tumour angiogenesis. J. Pathol..

[16] Mitra SK, Hanson DA, Schlaepfer DD (2005). Focal adhesion kinase: in command and control of cell motility. Nat. Rev. Mol. Cell. Biol..

[17] Parsons JT (2003). Focal adhesion kinase: the first ten years. J. Cell. Sci..

[18] Zhao J, Guan JL (2009). Signal transduction by focal adhesion kinase in cancer. Cancer Metastas. Rev..

[19] Kostourou V (2013). FAK-heterozygous mice display enhanced tumour angiogenesis. Nat. Commun..

[20] Golubovskaya V (2014). Targeting FAK in human cancer: from finding to first clinical trials. Front. Biosci..

[21] Schultze A, Fiedler W (2010). Therapeutic potential and limitations of new FAK inhibitors in the treatment of cancer. Expert Opin. Investig. Drugs.

[22] Sulzmaier FJ, Jean C, Schlaepfer DD (2014). FAK in cancer: mechanistic findings and clinical applications. Nat. Rev. Cancer.

[23] Tavora B (2010). Endothelial FAK is required for tumour angiogenesis. EMBO Mol. Med..

[24] Hirt UA (2018). Efficacy of the highly selective focal adhesion kinase inhibitor BI 853520 in adenocarcinoma xenograft models is linked to a mesenchymal tumor phenotype. Oncogenesis.

[25] Waldmeier L, Meyer-Schaller N, Diepenbruck M, Christofor G (2012). Py2T murinebreast cancer cells, a versatile model of TGF beta-induced EMT in vitro and in vivo. PLoS ONE.

[26] Shapiro IM (2014). Merlin deficiency predicts FAK inhibitor sensitivity: a synthetic lethal relationship. Sci. Transl. Med.

[27] Lin EY (2003). Progression to malignancy in the polyoma middle T oncoprotein mouse breast cancer model provides a reliable model for human diseases. Am. J. Pathol..

[28] Aslakson CJ, Miller FR (1992). Selective events in the metastatic process defined by analysis of the sequential dissemination of subpopulations of a mouse mammary tumor. Cancer Res..

[29] Lehembre F (2008). NCAM-induced focal adhesion assembly: a functional switch upon loss of E-cadherin. EMBO J..

[30] Hirt, U. A., et al. (eds). Abstract A249: BI 853520, a potent and highly selective inhibitor of protein tyrosine kinase 2 (focal adhesion kinase), shows efficacy in multiple xenograft models of human cancer. In *Proc. AACR-NCI-EORTC International Conference: Molecular Targets and Cancer Therapeutics* 12-16.11.2011 (San Francisco, CA, Philadelphia, 2011).

[31] Hirt UA, Haslinger C, Schweifer N, Garin-Chesa P, Adolf GR (2012). 469: E-cadherin expression predicts response of carcinomas to treatment with PTK2 inhibitors. Eur. J. Cancer.

[32] Canel M, Serrels A, Frame MC, Brunton VG (2013). E-cadherin-integrin crosstalk in cancer invasion and metastasis. J. Cell. Sci..

[33] Shapiro IM (2014). Merlin deficiency predicts FAK inhibitor sensitivity: a synthetic lethal relationship. Sci. Transl. Med..

[34] Chao YL, Shepard CR, Wells A (2010). Breast carcinoma cells re-express E-cadherin during mesenchymal to epithelial reverting transition. Mol. Cancer.

[35] Cicchini C (2008). TGFbeta-induced EMT requires focal adhesion kinase (FAK) signaling. Exp. Cell Res..

[36] Hugo H (2007). Epithelial–mesenchymal and mesenchymal–epithelial transitions in carcinoma progression. J. Cell. Physiol..

[37] van Nimwegen MJ, Verkoeijen S, van Buren L, Burg D, van de Water B (2005). Requirement for focal adhesion kinase in the early phase of mammary adenocarcinoma lung metastasis formation. Cancer Res..

[38] Wendt MK, Schiemann WP (2009). Therapeutic targeting of the focal adhesion complex prevents oncogenic TGF-beta signaling and metastasis. Breast Cancer Res..

[39] Wendt MK, Taylor MA, Schiemann BJ, Schiemann WP (2011). Down-regulation of epithelial cadherin is required to initiate metastatic outgrowth of breast cancer. Mol. Biol. Cell..

[40] Shibue T, Brooks MW, Inan MF, Reinhardt F, Weinberg RA (2012). The outgrowth of micrometastases is enabled by the formation of filopodium-like protrusions. Cancer Discov..

[41] Ward KK (2013). Inhibition of focal adhesion kinase (FAK) activity prevents anchorage-independent ovarian carcinoma cell growth and tumor progression. Clin. Exp. Metastas..

[42] Mitra SK, Lim ST, Chi A, Schlaepfer DD (2006). Intrinsic focal adhesion kinase activity controls orthotopic breast carcinoma metastasis via the regulation of urokinase plasminogen activator expression in a syngeneic tumor model. Oncogene.

[43] Wu ZM, Yuan XH, Jiang PC, Li ZQ, Wu T (2006). Antisense oligonucleodes targeting the focal adhesion kinase inhibit proliferation, induce apoptosis and cooperate with cytotoxic drugs in human glioma cells. J. Neurooncol..

[44] Smith CS (2005). Effect of focal adhesion kinase (FAK) downregulation with FAK antisense oligonucleotides and 5-fluorouracil on the viability of melanoma cell lines. Melanoma Res..

[45] Chen Y (2010). The effect of focal adhesion kinase gene silencing on 5-fluorouracil chemosensitivity involves an Akt/NF-jB signaling pathway in colorectal carcinomas. Int. J. Cancer.

[46] van Nimwegen MJ, Huigsloot M, Caier A, Tijdens IB, van de Water B (2006). Focal adhesion kinase and protein kinase B cooperate to suppress doxorubicin-induced apoptosis of breast tumor cells. Mol. Pharmacol..

[47] Halder J (2006). Focal adhesion kinase targeting using in vivo short interfering RNA delivery in neutral liposomes for ovarian carcinomatherapy. Clin. Cancer Res..

[48] Zhang HM (2006). Induced focal adhesion kinase expression suppresses apoptosis by activating NF-kB signaling in intestinal epithelial cells. Am. J. Physiol..

[49] Huanwen W (2009). Intrinsic chemoresistance to gemcitabine is associated with constitutive and laminin-induced phosphorylation of FAK in pancreatic cancer cell lines. Mol. Cancer.

[50] Duxbury MS (2003). RNA interference targeting focal adhesion kinase enhances pancreatic adenocarcinoma gemcitabine chemosensitivity. Biochem. Biophys. Res. Commun..

[51] Halder J (2007). Therapeutic efficacy of a novel focal adhesion kinase inhibitor TAE226 in ovarian carcinoma. Cancer Res..

[52] Hochwald SN (2009). A novel small molecule inhibitor of FAK decreases growth of human pancreatic cancer. Cell Cycle.

[53] Kang Y (2013). Role of focal adhesion kinase in regulating YB-1-mediated paclitaxel resistance in ovarian cancer. J. Natl. Cancer Inst..

[54] Symeonides SN, Anderton SM, Serrels A (2017). FAK-inhibition opens the door to checkpoint immunotherapy in pancreatic cancer. J. Immunother. Cancer.

[55] Jiang H (2016). Targeting focal adhesion kinase renders pancreatic cancers responsive to checkpoint immunotherapy. Nat. Med..

[56] Serrels A (2015). Nuclear FAK controls chemokine transcription, Tregs, and evasion of anti-tumor immunity. Cell.

[57] Tavora B (2014). Endothelial-cell FAK targeting sensitizes tumours to DNA-damaging therapy. Nat. Lett..

[58] Cooke VG (2012). Pericyte depletion results in hypoxia-associated epithelial-to-mesenchymal transition and metastasis mediated by met signaling pathway. Cancer Cell.

[59] Clarke RB, Stingl J, Vivanco M, Bentires-Alj M (2013). “The charmingest place”: non-coding RNA, lineage tracing, tumor heterogeneity, metastasis and metabolism—new methods in mammary gland development and cancer: the fifth ENBDC Workshop. Breast Cancer Res..

[60] Fantozzi A, Christofori G (2006). Mouse models of breast cancer metastasis. Breast Cancer Res..

[61] Diepenbruck M (2017). miR-1199-5p and Zeb1 function in a double-negative feedback loop potentially coordinating EMT and tumour metastasis. Nat. Commun..

[62] Dobin A (2013). STAR: ultrafast universal RNA-seq aligner. Bioinformatics..

[63] Gaidatzis D, Lerch A, Hahne F, Stadler MB (2015). QuasR: quantification and annotation of short reads in R. Bioinformatics.

[64] Robinson MD, McCarthy DJ, Smyth GK (2010). edgeR: a Bioconductor package for differential expression analysis of digital gene expression data. Bioinformatics.

[65] Gene Ontology C. (2015). Gene Ontology Consortium: going forward. Nucleic Acids Res..

[66] Falcon S, Gentleman R (2007). Using GOstats to test gene lists for GO term association. Bioinformatics.

[67] Yu G, He QY (2016). ReactomePA: an R/Bioconductor package for reactome pathway analysis and visualization. Mol. Biosyst..

