# Supplementary material for: The FAK inhibitor BI 853520 exerts anti-tumor effects in breast cancer
Source: Oncogenesis. 2018 Sep 20;7(9):73. doi: 10.1038/s41389-018-0083-1 (PMC6148276; doi:10.1038/s41389-018-0083-1)
Supplement: Supplementary file 1 — Supplementary Material [file 41389_2018_83_MOESM1_ESM.pdf]

Supplemental information

The FAK-Inhibitor BI 853520 exerts anti-tumor effects in breast cancer

Stefanie Tiede, Nathalie Meyer-Schaller, Ravi Kiran Reddy Kalathur, Robert Ivanek, Ernesta Fagiani, Philip Schmassmann, Patrick Stillhard, Simon Häfliger, Norbert Kraut, Norbert Schweifer, Irene C. Waizenegger, Ruben Bill and Gerhard Christofori

Supplementary Figures

Supplementary Figure 1

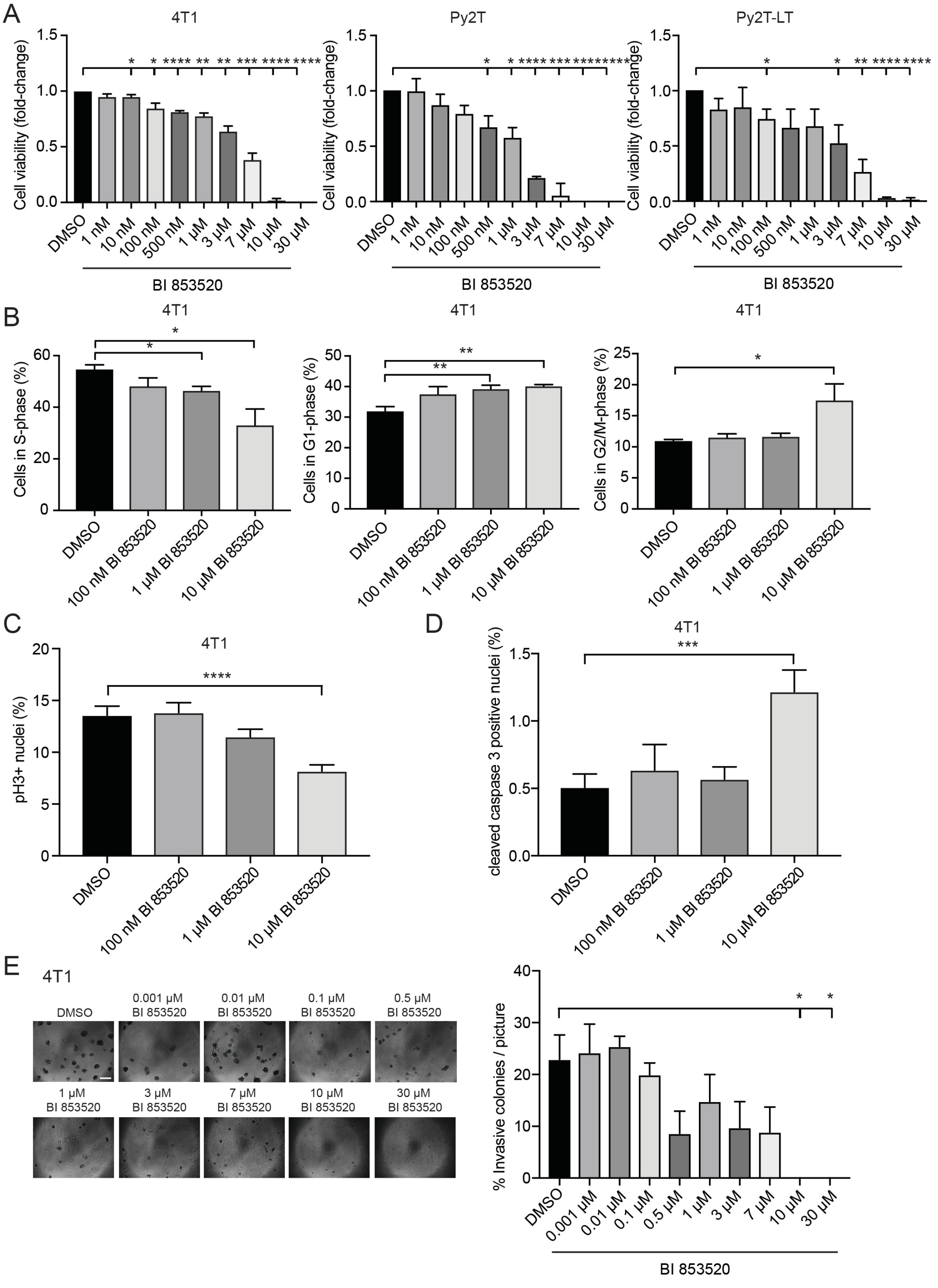

Supplementary Figure 1. Anti-proliferative effects of BI 853520 on 4T1 murine breast cancer cells *in vitro*.

(A) Left panel: 4T1 breast cancer cells were exposed to increasing concentrations of BI 853520 one day after cell seeding and cell viability was measured at day five (n = 3). Middle panel: Py2T breast cancer cells were exposed to increasing concentrations of BI 853520 one day after cell seeding and cell viability was measured at day five (n = 3). Right panel: Py2T-LT breast cancer cells were exposed to increasing concentrations of BI 853520 one day after cell seeding and cell viability was measured at day five (n = 3).

(B) 4T1 cells were treated for 24 hours with varying concentrations of BI 853520, incubated for five minutes with 10µM EdU and then subjected to EdU/PI cell cycle flow cytometry analysis as described in Materials and Methods (n = 4). Cell numbers in S, G1 and G2/M-phases were quantified, the results are depicted in the three panels.

(C) 4T1 breast cancer cells were treated for 24 hours with varying concentrations of BI 853520 and then immunostained for phospho-histone 3 (pH3) and DAPI (nuclei). Quantification of the percentage of pH3-positive nuclei is shown, indicating a decrease in 4T1 cell proliferation (n = 4).

(D) 4T1 breast cancer cells were treated for 24 hours with varying concentrations of BI 853520 and then immunostained for cleaved caspase-3-positive nuclei. Quantification of the cleaved caspase-3-positive cells is shown, indicating no change in the rate of cell apoptosis except at the non-specific concentration of 10µM (n = 4). Statistical analyses were performed using an unpaired, two-tailed Student's t test.

All data are depicted as mean ± SEM. ****, $p < 0.0001$; ***, $p < 0.001$; **, $p < 0.01$; *, $p < 0.05$.

(E) BI 853520 represses 4T1 breast cancer cell invasion cultured in Matrigel. 4T1 breast cancer cells were seeded in Matrigel and treated with increasing concentrations of BI 853520 as indicated. Phase contrast microscopy pictures were taken and the numbers of invasive colonies per picture were quantified (n = 1). Statistical analysis was performed using an unpaired, two-tailed Student's t test. ****, $p < 0.0001$; ***, $p < 0.001$; **, $p < 0.01$; *, $p < 0.05$. Scale bar, 250µm.

Supplementary Figure 2

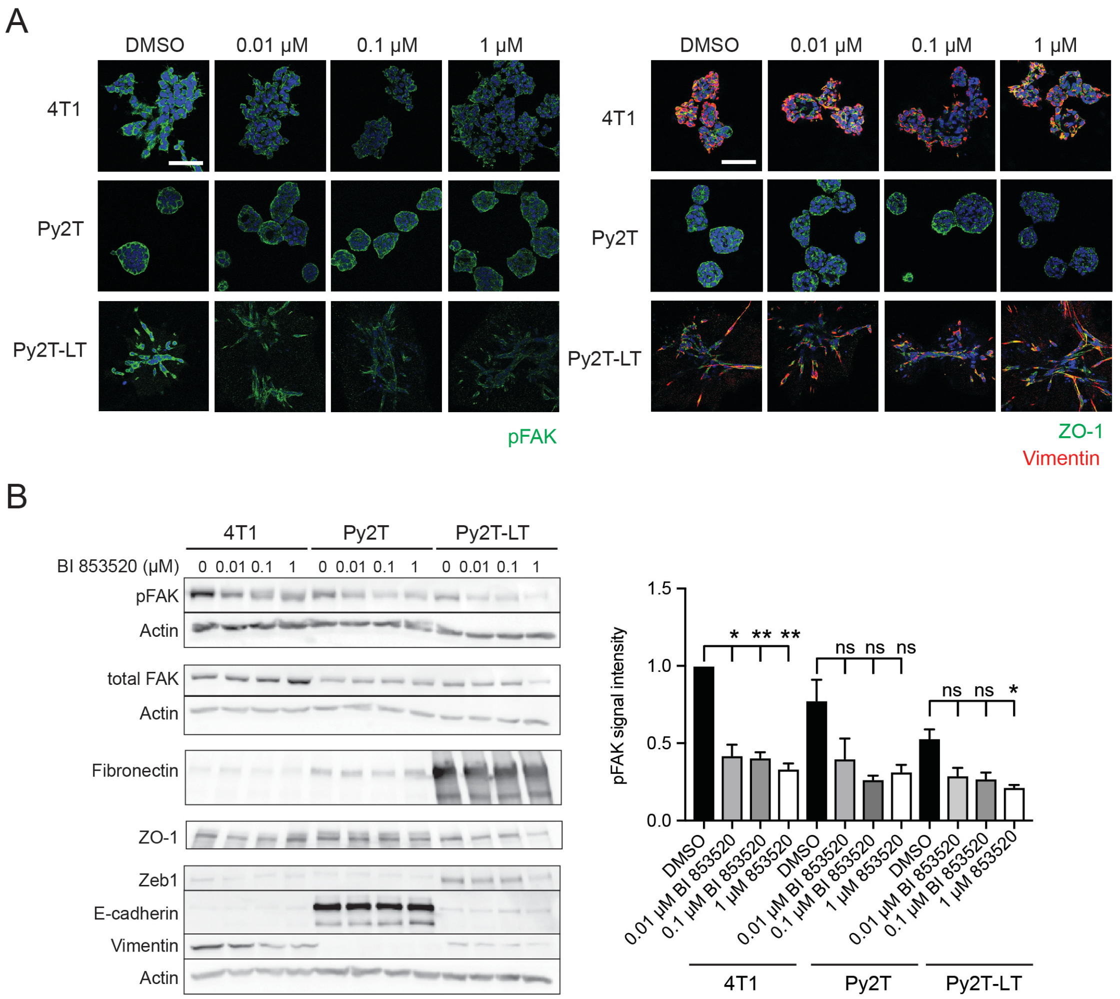

Suppl. Figure 2. BI 853520 decreases Y397-FAK phosphorylation in 3D Matrigel cultures in a dose-dependent manner.

(A) 4T1, Py2T and Py2T-LT cells grown in a 3D Matrigel culture (4mg/ml) were treated with increasing concentrations of BI 853520 (0.01µM, 0.1µM, 1µM) for 24 hours, followed by immunofluorescence staining for pFAK (Y397, green) and DNA (blue) (left panel). The 3D cultured cells were also stained for ZO-1 (green), vimentin (red) and DNA (blue) (right panel). Images were obtained using a SP5 laser-scanning confocal microscope (Leica) (n = 2). Scale bars, 30µm.

(B) 4T1, Py2T and Py2T-LT cells grown in a 5% 3D Matrigel culture were exposed to increasing concentrations of BI 853520 (0.01µM, 0.1µM, 1µM) for 24 hours (n = 2). Left panel: Cell lysates were examined by immunoblotting analysis for phospho-FAK-Y397 (pFAK) and total FAK (FAK), fibronectin, ZO-1, Zeb1, E-cadherin and vimentin. Actin was used as a loading control. Right panel: Quantification of the pFAK signal

intensities normalized to actin and total FAK/actin ratios from the immunoblotting analysis shown in the left panel. Statistical analysis was performed using an unpaired, two-tailed Student's t test. ****, p < 0.0001; ***, p < 0.001; **, p < 0.01; *, p < 0.05.

Supplementary Figure 3

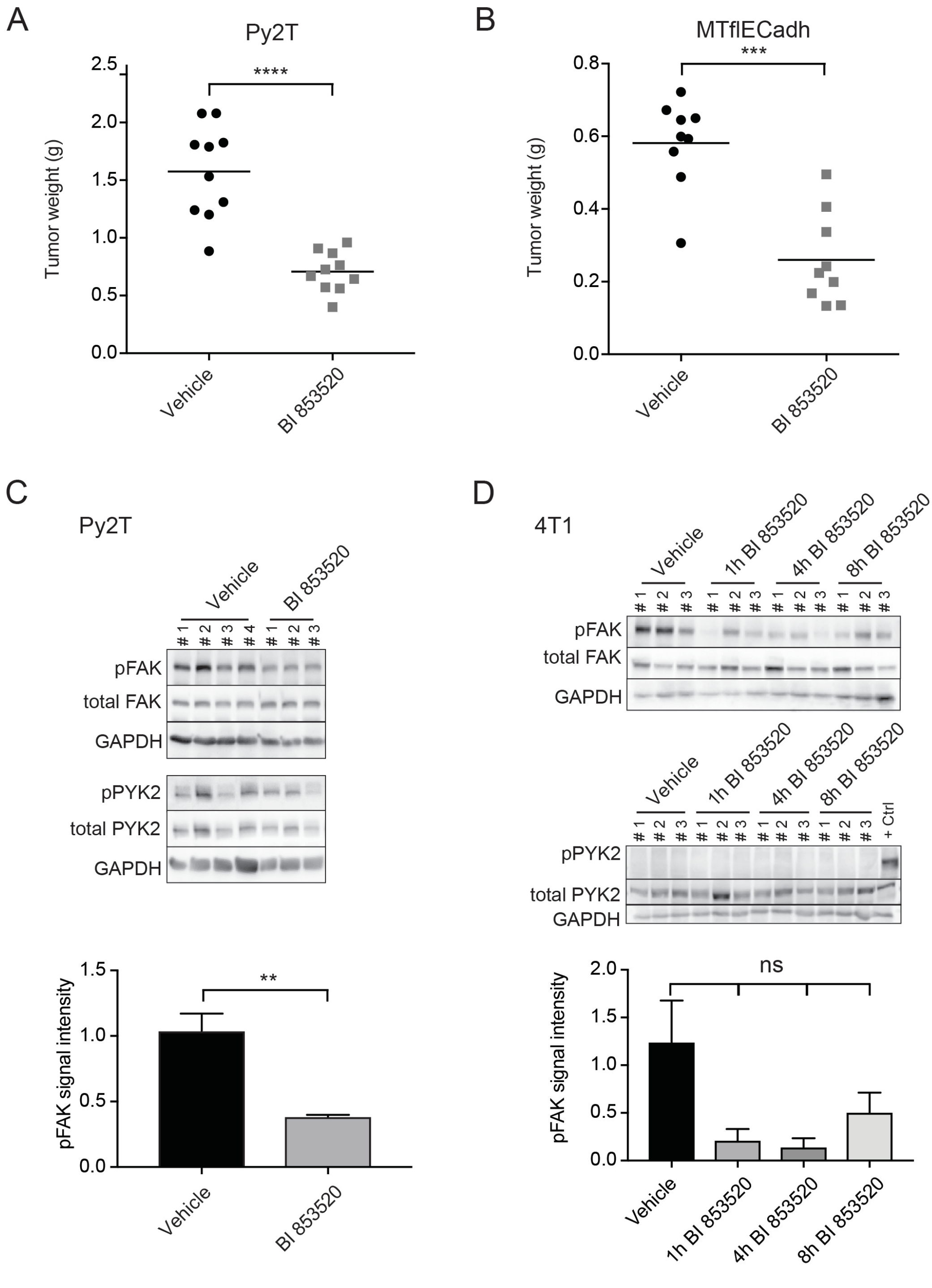

Supplementary Figure 3. BI 853520 reduces primary tumor in different preclinical breast cancer mouse models and delays malignant tumor progression.

(A) Treatment of mice bearing orthotopically transplanted Py2T breast tumors with 50mg/kg BI 853520 daily for 25 days significantly decreases tumor weight at the experimental end point. n = 10 mice per treatment cohort. Statistical analysis was performed by the unpaired, two-tailed Mann-Whitney U test.

(B) Treatment of mice bearing orthotopically transplanted MTflECad breast tumors with 50 mg/kg BI 853520 daily significantly decreases tumor weight. n = 9 mice per treatment group. Statistical analysis was performed by the unpaired, two-tailed Mann-Whitney U test.

(C) Three-day daily 50mg/kg BI 853520-treated Py2T primary tumor pieces were snap-frozen 20h post last treatment. Top panel: Lysates were analyzed by immunoblotting analysis for phospho-FAK (pFAK, Y397), total FAK, phospho-PYK2 (pPYK2, Y402), total PYK2 and GAPDH as loading control. Bottom panel: Quantification of pFAK signal intensity normalized to GAPDH and total FAK/GAPDH ratios from the immunoblotting analysis shown above. Statistical analysis was performed using an unpaired, two-tailed Student's t test. ****, $p < 0.0001$; ***, $p < 0.001$; **, $p < 0.01$; *, $p < 0.05$.

(D) Single dose 50mg/kg BI 853520-treated 4T1 primary tumor pieces were snap-frozen 1, 4 and 8 hours post treatment. Top panel: Lysates were analyzed by immunoblotting analysis for phospho-FAK (pFAK, Y397), total FAK, phospho-PYK2 (pPYK2, Y402), total PYK2 and GAPDH as loading control. Bottom panel: Quantification of pFAK signal intensity normalized to GAPDH and total FAK/GAPDH ratios from the immunoblotting analysis shown above. Statistical analysis was performed using an unpaired, two-tailed Student's t test. ****, $p < 0.0001$; ***, $p < 0.001$; **, $p < 0.01$; *, $p < 0.05$.

Supplementary Figure 4

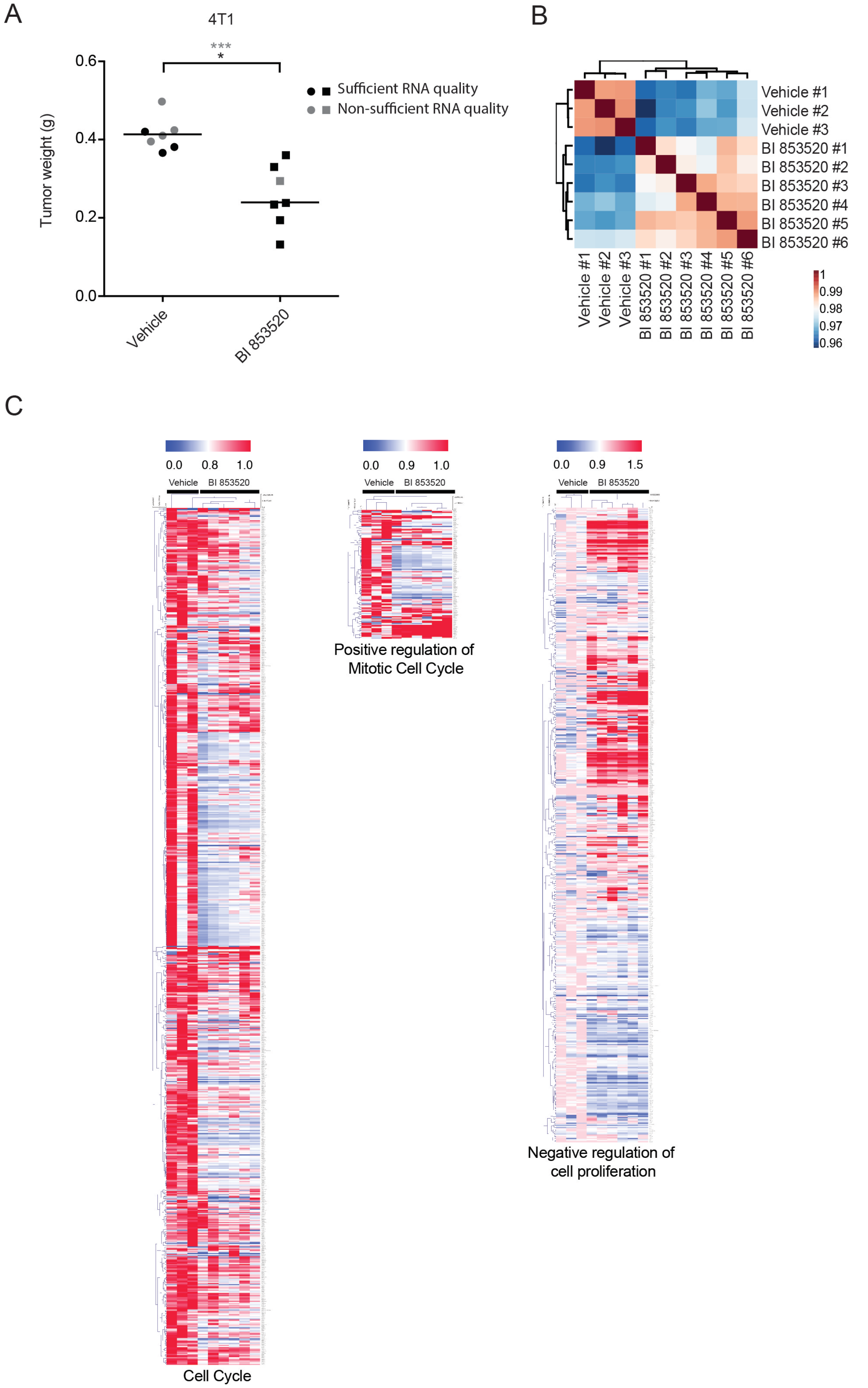

Supplementary Figure 4. Gene expression profiling reveals that BI 853520 represses murine breast cancer cell proliferation.

(A) BALB/c mice were implanted with 4T1 murine breast cancer cells and once tumors had formed treated for 5 days with vehicle control or with 50mg/kg BI 853520 daily. Tumor volumes are shown after treatment period. RNA was then extracted from primary tumors, and RNA samples with adequate quality were subjected to RNA sequencing and gene expression profiling analysis (indicated with black dots/rectangles). Statistical analysis was performed using an unpaired, two-tailed Mann-Whitney U test of all tumors (grey asterisks) and separately of only the tumors with sufficient RNA-quality (black asterisk; n = 7, tumors in the control group; n = 7, tumors in the BI 853520 -treated group). ****, $p < 0.0001$; ***, $p < 0.001$; **, $p < 0.01$; *, $p < 0.05$.

(B) Correlation analysis of gene expression data obtained from RNA-sequencing followed by hierarchical clustering showed a distinct grouping of vehicle and BI 853520-treated primary tumors. The color codes represent correlation values.

(C) Heatmaps of genes with changes in gene expression after BI 853520 treatment: the terms “general cell cycle”, “positive regulation of mitotic cell cycle” and “negative regulation of cell proliferation” were identified by gene set enrichment analysis.

Supplementary Figure 5

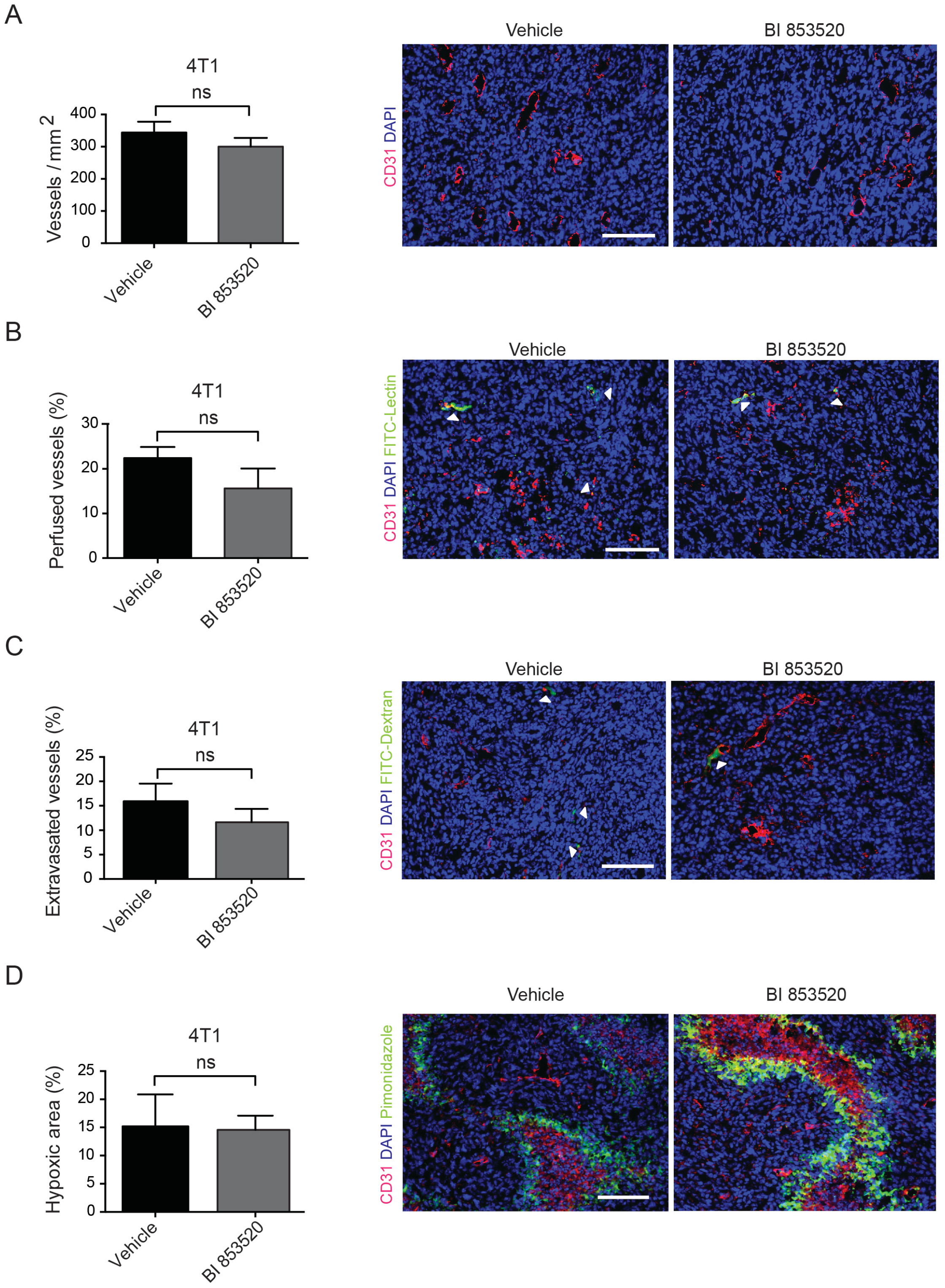

Supplementary Figure 5. BI 853520 does not regulate angiogenesis in a relevant manner *in vivo*.

(A) Immunofluorescence microscopy analysis of primary tumor sections of 4T1 breast tumor bearing mice treated with vehicle control or 50mg/kg daily BI 853520 and analyzed at the experimental end-point for DAPI (cell nuclei; blue) and CD31 (red). Quantification of the numbers of vessels per mm^2 is shown on the left, representative immunofluorescence images are shown on the right. n = 7 tumors in the vehicle group, n = 8 tumors in the BI 853520 treated group.

(B) Immunofluorescence microscopy analysis of primary tumor sections of 4T1 breast tumor bearing mice treated with vehicle control or 50mg/kg daily BI 853520 and analyzed at the experimental end-point for DAPI (cell nuclei; blue) and CD31 (red). Perfused vessels were FITC-lectin positive (green). Quantification of the percentage of lectin-positive vessels is shown in the left panel, representative immunofluorescence images are shown on the right. n = 2 tumors per group.

(C) Immunofluorescence staining of primary tumor sections of 4T1 breast tumor bearing mice treated with vehicle control or 50mg/kg daily BI 853520 and analyzed at the experimental end-point for DAPI (cell nuclei; blue) and CD31 (red). Leaky vessels showed extravasated FITC-Dextran (green) in their vicinity. Quantification of the percentage of Dextran-positive vessels is shown in the left panel, representative immunofluorescence images are shown on the right. n = 2 tumors in the vehicle group, n = 3 tumors in the BI 853520 treated group.

(D) Immunofluorescence staining of primary tumor sections of the 4T1 breast tumor bearing mice treated with vehicle control or 50mg/kg daily BI 853520 and analyzed at the experimental end-point for DAPI (cell nuclei; blue), CD31 (red) and pimonidazole (green). Quantification of the percentage hypoxic area is shown in the left panel, representative immunofluorescence images are shown on the right. n = 4 tumors in the control group, n = 5 tumors in the BI 853520 treated group (two sections per tumor were analyzed).

Data indicate counts per field of view, shown as mean ± SEM. Statistical difference was determined by the unpaired, two-tailed Student's t test. ****, $p < 0.0001$; ***, $p < 0.001$; **, $p < 0.01$; *, $p < 0.05$. Scale bars, 100μm.

Supplementary Figure 6

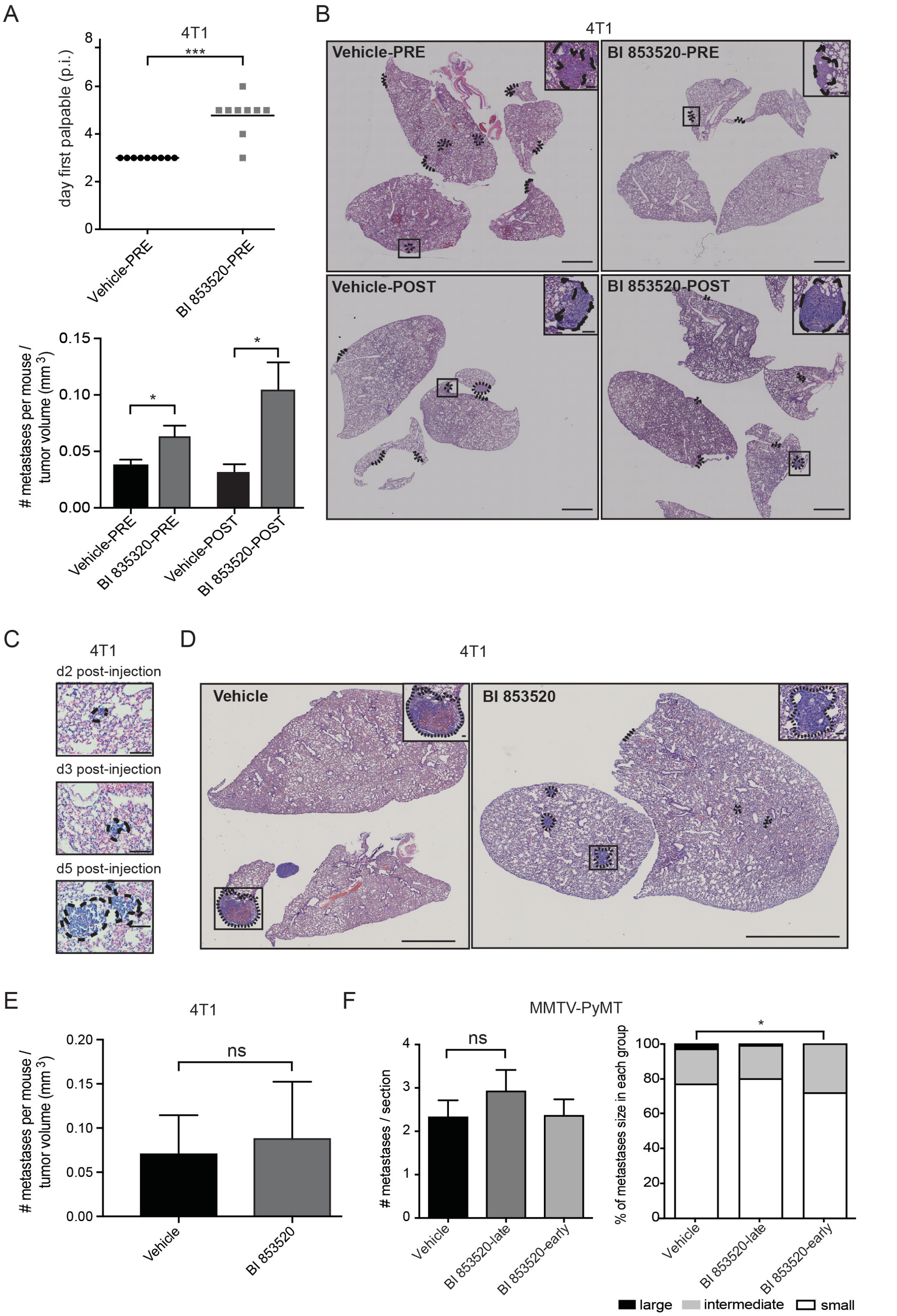

Supplementary Figure 6. BI 853520 reduces pulmonary metastasis outgrowth.

(A) Tumor onset is significantly delayed in s.c. tumors pre-treated daily with 50mg/kg BI 853520 (palpable median five days post tumor cell injection) compared to vehicle pre-treated cohort (palpable three days post tumor cell injection). n = 9, Vehicle-PRE; n = 9, BI 853520-PRE. The ratio between the number of metastasis per mouse to the primary tumor volume represents the normalized metastatic index. Statistical analysis was performed using an unpaired, two-tailed Mann-Whitney U test.

(B) Mice were either pre-treated daily starting three days prior to 4T1 tumor cell inoculation (Vehicle-PRE or BI 853520-PRE; 50mg/kg BI 853520) or treated seven days post tumor cell injection (Vehicle-POST or BI 853520-POST groups) as described in Figure 5. Representative H&E staining of pulmonary histological sections are shown. Dashed lines indicate metastatic lesions. Scale bars, 2mm; scale bars in zoom-in, 100µm.

(C) Representative images of H&E-stained lungs following intravenous 4T1 injection. Cells appear to start proliferating three days post intravenous injection. Dashed lines indicate metastatic lesions. Scale bar, 100µm.

(D) Representative images of H&E stained pulmonary cross-sections of adjuvant BI 853520 therapy. Dashed lines indicate metastatic lesions. Scale bar, 2mm, scale bars in zoom-in, 100µm.

(E) Normalization of the number of metastasis to primary tumor growth (metastatic index) in mice transplanted with 4T1 cells and treated daily with 50mg/kg BI 853520 in a neoadjuvant setting until primary tumors were removed and therapy was discontinued. Numbers of metastasis were determined 18 days after primary tumor removal (see also Fig. 5D). The ratio between the number of metastasis per mouse to the primary tumor volume represents the normalized metastatic index. Statistical analysis by unpaired, two-tailed Student's t-test.

(F) BI 853520 therapy has no effect on the number of pulmonary metastases in the MMTV-PyMT transgenic mouse model treated with vehicle, late BI 853520 therapy or early BI 853520 therapy. n = 9, Vehicle; n = 9, BI 853520-late; n = 8, BI 853520-early (see Fig. 2D-E). Left panel: Numbers of metastases per section were quantified. Right panel: Quantification of the percentage of small, medium and large size individual lung metastatic lesions. Statistical analysis was performed using a Fisher’s exact test. All data are depicted as mean ± SEM. ****, $p < 0.0001$; ***, $p < 0.001$; **, $p < 0.01$; *, $p < 0.05$.
